# The accessory protein MRAP2 directly interacts with melanocortin-3 receptor to enhance signaling

**DOI:** 10.1126/scisignal.adu4315

**Published:** 2025-12-16

**Authors:** Aqfan Jamaluddin, Rachael A. Wyatt, Joon Lee, Georgina K.C. Dowsett, John A. Tadross, Johannes Broichhagen, Giles S.H. Yeo, Joshua Levitz, Caroline M. Gorvin

**Affiliations:** 1 Department of Metabolism and Systems Science, University of Birmingham, Birmingham, B15 2TT, UK; 2 Centre of Membrane Proteins and Receptors (COMPARE), Universities of Birmingham and Nottingham, Birmingham, B15 2TT, UK; 3 Department of Biochemistry, Weill Cornell Medicine, New York, NY 10065, USA; 4 Wellcome-MRC Institute of Metabolic Science-Metabolic Research Laboratories, University of Cambridge, Cambridge, CB2 0QQ, UK; 5 East Genomics Laboratory Hub, Cambridge University Hospitals NHS Foundation Trust, Cambridge, CB2 0QQ, UK; 6 Department of Histopathology, Cambridge University Hospitals NHS Foundation Trust, Cambridge, CB2 0QQ, UK; 7 Leibniz-Forschungsinstitut für Molekulare Pharmakologie (FMP), 13125, Berlin, Germany

## Abstract

The central melanocortin system links nutrition to energy expenditure. Melanocortin-4 receptor (MC4R) controls appetite and food intake, and its signaling is potentiated by melanocortin-2 receptor accessory protein-2 (MRAP2). Human mutations in *MC4R* and *MRAP2* are associated with obesity. Here, we sought to determine whether MRAP2 affected the activity of MC3R, which is structurally similar to MC4R and which regulates sexual maturation, linear growth rate, and lean mass accumulation. Single-molecule pull-down assays showed that MC3R and MRAP2 interacted in HEK293 cells. Analysis of fluorescent photobleaching steps showed that MC3R and MRAP2 readily formed heterodimers, most commonly with a 1:1 stoichiometry. Mining of previously published human single-nucleus and spatial transcriptomic data showed coexpression of *MRAP2* and *MC3R* in hypothalamic neurons that function in energy homeostasis and appetite control. In HEK293 cells, MRAP2 enhanced cAMP signaling downstream of MC3R, impaired β-arrestin recruitment to MC3R, and reduced MC3R internalization. The ability of MRAP2 to promote MC3R signaling was suppressed by alanine mutagenesis of five MRAP2 and two MC3R transmembrane residues identified by structural homology models as important for the interaction. We showed that variants of MRAP2 found in individuals who are overweight or obese did not enhance MC3R-driven signaling. Thus, these studies implicate MRAP2 as an important regulator of MC3R function and provide further evidence for the crucial role of MRAP2 in energy homeostasis.

## Introduction

The melanocortin receptor-2 accessory protein 2 (MRAP2) is a single-pass transmembrane protein that modulates the function of several G protein-coupled receptors (GPCRs) expressed in the hypothalamus that regulate food intake (1–4). These GPCRs include melanocortin receptor-4 (MC4R), a central regulator of appetite, inactivating mutations of which are the most common genetic cause of obesity, and the receptor for ghrelin (growth hormone secretagogue receptor, GHSR), which enhances appetite (1, 2, 4). Similarly to MC4R, human genetic variants in *MRAP2* have been identified in several families and individuals with obesity that reduce MC4R activity (5–7). MRAP2 was identified as a homolog of MRAP1, an accessory protein that is essential for the cell surface expression and ligand responsiveness of melanocortin receptor-2 (MC2R), which regulates adrenal development and steroidogenesis (2). Unlike MRAP1, MRAP2 is not essential for GPCR expression at the cell surface. However, MRAP2 enhances MC4R expression at neuronal primary cilia, a microtubule-based organelle involved in appetite regulation (8), suggesting that MRAP2 may establish signaling hubs that favor receptor signaling.

Deletion of the *MRAP2* gene from mice on various genetic backgrounds is associated with extreme obesity, increased fat mass and visceral adiposity, analogous to *MC4R* knockout mice (9, 10). Analysis of mice deficient in both *MRAP2* and *MC4R* demonstrate that MRAP2 facilitates the action of MC4R but that there are also MC4R-independent mechanisms (5). *MRAP2* knockout mice lack the early-onset hyperphagia of *MC4R* knockout mice, and humans with *MRAP2* genetic variants exhibit hyperglycaemia, hypertension and high blood cholesterol more frequently than those with *MC4R* mutations (6). These effects are consistent with previous work showing that MRAP2 can modulate the signaling profile of several GPCRs involved in energy homeostasis. MRAP2 enhances signaling by MC4R and the ghrelin receptor, whereas it suppresses the activity of the prokineticin receptors (3), orexin receptor-1 (11) and melanin concentrating hormone receptor-1 (12). One study identified >40 putative binding partners for MRAP2 (13); however, signaling data was not provided for most receptors, and some had previously been described as non-interacting proteins, and therefore further work is required to validate these findings. Additionally, although MC4R signaling is impaired by some MRAP2 variants identified in overweight or obese individuals (6, 7, 14, 15), their effect on signaling by other MRAP2 interacting proteins remains to be explored.

MRAP2 can interact with all five members of the melanocortin receptor family when overexpressed in cell lines (2, 16). MC3R is an inhibitor of the central melanocortin system (17, 18). It is required for the normal activation of AgRP neurons in response to nutritional deficit (17). *MRAP2* deletion from AgRP neurons also blunts their fasting-induced activation (1), similarly to that of *MC3R* deletion, and it has been hypothesized that a complex signaling system may exist between MC3R, MRAP2 and other receptors at these neurons (17). There is some inconclusive evidence that MC3R may interact with MRAP2. MRAP2 coimmunoprecipitates with MC3R (2) and enhances MC3R expression in cilia in transfected cells (8). However, co-expression of MC3R and MRAP2 can reduce (2), enhance (5), or not affect (19, 20) cAMP signaling, depending on the study. Thus, further examination of the effect of MRAP2 on MC3R activity is warranted. Such inconsistencies are common in the MRAP2 literature, with MRAP2 initially described to reduce MC4R cell surface expression and impair its signaling, then later shown to increase MC4R function, consistent with mouse knockout studies (2, 3, 13). These discrepancies may be due to large experimental variations in the investigation of MRAP2 function, such as overexpressing MRAP2 at DNA ratios of 3-20x that of the GPCR (11–13, 21). As such, these high concentrations of MRAP2 could lead to overexpression artefacts and false positive results (22).

MRAP2 facilitates signaling by some GPCRs (5, 21) and suppresses responses by other receptors (3, 23). MRAP2 may enhance signaling by reducing the ability of the ghrelin receptor (GHSR) (21), prokineticin receptor-2 (24) and MC4R (21) to recruit β-arrestin proteins, sometimes without changes in receptor cell surface expression. Additionally, MRAP2 biases GHSR signaling to reduce Rho activation, enhances G protein coupling of MC4R, and may reduce MC4R oligomerization that can suppress receptor signaling (21, 23). The structural regions involved in MRAP2 interaction with GPCRs remain largely unexplored. MC4R homology models based on the cryo-EM structure of the MC2R-MRAP1 complex suggest that MRAP2 may interact with transmembrane helix 5 (TM5) or TM6, although mechanistic insight is missing (21). Additionally, although MRAP2 mutants with large truncations (such as deletion of the transmembrane region or deletion of the C-tail) show loss of interaction or impaired signaling (11), these mutants do not provide insights into the specific residues involved or their mechanisms of action.

Here, we examined the effect of MRAP2 on MC3R activity in HEK293 cells. We demonstrated that MRAP2 interacted with MC3R in a 1:1 dimer to enhance cAMP signaling, reduce β-arrestin recruitment, and impair receptor internalization. Structural homology models and alanine mutagenesis identified critical residues important for the interaction. Finally, we demonstrated that MRAP2 variants identified in individuals who are overweight or obese reduced MC3R signaling and enhanced receptor internalization.

## Results

### *MRAP2* is colocalised with *MC3R* in neurons involved in energy homeostasis

**Fig. 1:**
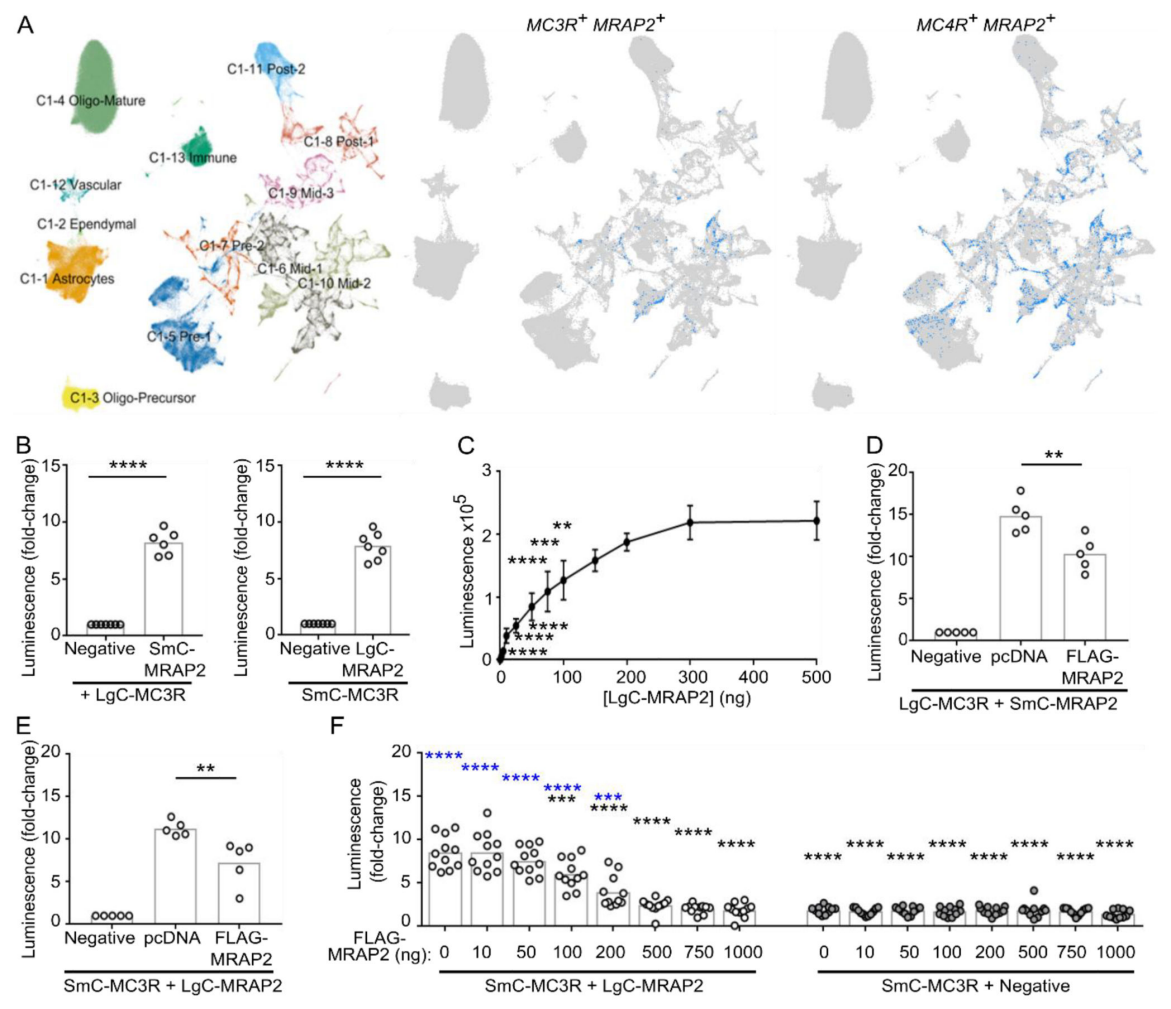
*MC3R* is coexpressed with *MRAP2* in hypothalamic neurons (**A**) snRNA-seq analysis of the human hypothalamus for *MRAP2, MC3R*, and *MC4R* from (25). (Left) UMAP plot of the snRNA-seq data from HYPOMAP, with cells coloured by C1 clustering. (Middle) UMAP plot highlighting cells in blue that coexpress *MRAP2* and *MC3R*. (Right) UMAP plot highlighting cells in blue that coexpress *MRAP2* and *MC4R* transcripts. Table S1 shows the top 15 clusters with the highest *MC3R* or *MC4R* expression, with the percentage coexpression of *MRAP2* in each cluster. (**B**) NanoBiT luminescence between MC3R and MRAP2 or negative control (MC3R-SmC and LgC-Empty). N=6-7 biological replicates per group. (**C**) NanoBiT luminescence between 100ng SmC-MC3R and increasing concentrations of LgC-MRAP2. The negative control was MC3R-SmC and LgC-Empty. N=4 biological replicates per group. (**D-E**) Competition assays with NanoBiT constructs and 100 ng pcDNA or 100 ng MRAP2. N=5 biological replicates per group. Data are expressed relative to the negative control in (B) to (D). (**F**) Competition assays with NanoBiT constructs and increasing concentrations of MRAP2. N=11 biological replicates per group. Data are expressed relative to Sm-C-MC3R + Negative with 0 ng FLAG-MRAP2. **p<0.01, ***p<0.001, ****p<0.0001 by unpaired student’s t-test in (B) and one-way ANOVA with Dunnett’s test in (C) to (E). Black asterisks compare responses to SmC-MC3R + LgC-MRAP2 + 0 ng FLAG-MRAP2. Blue asterisks compare responses to SmC-MC3R + Negative + 0 ng FLAG-MRAP2.

It has been unclear whether MRAP2 interacts with MC3R to influence receptor signaling (2, 5, 19, 20). Because coexpression in the same cells is a requirement for biologically relevant MC3R-MRAP2 interactions, we first assessed expression of the transcripts encoding MC3R and MRAP2 proteins in HYPOMAP, a single-nucleus RNA-sequencing (snRNA-seq) and spatial transcriptomic atlas of the human hypothalamus (25). *MRAP2* is expressed in ~35% of all neuronal cells and is detected in 57% of *MC3R*-positive neurons, indicating that the two genes have overlapping coexpression in physiologically relevant cell types (Fig. 1A, table S1). By comparison, *MRAP2* is detected in 53% of *MC4R*-positive neurons in HYPOMAP (Fig. 1A, table S1). Visium spatial transcriptomics analysis revealed that *MRAP2* is expressed throughout the hypothalamus, particularly in regions where there is greater neuronal density, whereas *MC3R* expression is more restricted to the arcuate nucleus, ventromedial hypothalamus and periventricular region (fig. S1, table S1). *MRAP2* transcripts are present under the same spatially barcoded spots as *MC3R* transcripts in these regions, which are critical for energy homeostasis and appetite control.

### MRAP2 interacts with MC3R

To determine whether MC3R and MRAP2 are likely to interact, we first assessed protein proximity in transiently transfected HEK293 cells using the NanoBiT split-luciferase system with both proteins tagged at the C-terminus. There was increased luminescence observed in cells co-expressing MC3R and MRAP2 compared to cells expressing MC3R and the negative control (Fig.1B). Similar luminescence values were observed in cells transfected with either iteration of NanoBiT tags (specifically LgC-MC3R and SmC-MRAP2 or SmC-MC3R and LgC-MRAP2). Saturation curves with a fixed amount of MC3R (100 ng) transfected with increasing concentrations of MRAP2 resulted in a hyperbolic increase in the luminescence, indicating the signal was unlikely to be due to random collisions (Fig. 1C). Co-transfection of cells with 100 ng FLAG-MRAP2 to compete with SmC/LgC-MRAP2 reduced NanoBiT luminescence values (Fig. 1D-E), providing further evidence that the two proteins may interact. Concentrations of FLAG-MRAP2 at 100 ng and above disrupted MC3R-MRAP2 NanoBiT interactions, whereas concentrations of FLAG-MRAP2 at 500 ng and above fully displaced LgC-MRAP2 (Fig. 1F).

**Fig. 2:**
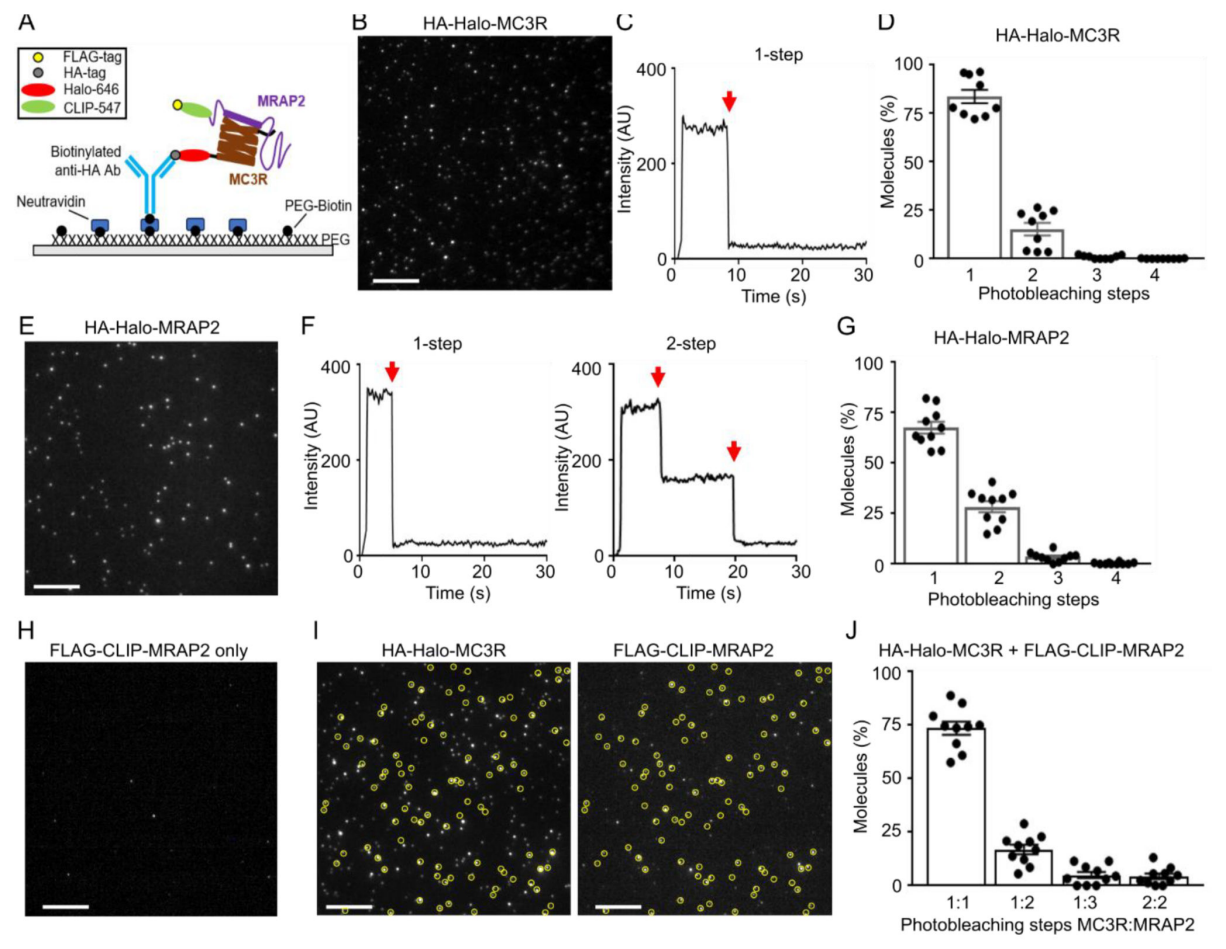
MC3R and MRAP2 interact primarily in a 1:1 stoichiometry (**A**) For two-color SiMPull experiments, freshly prepared lysate from HEK293 cells expressing HA-Halo-MC3R with FLAG-CLIP-MRAP2 was added to a PEG-passivated glass slide containing immobilized HA antibody. Halo and CLIP tags were labeled with CA-Sulfo646 and BC-DY547, respectively. (**B** and **C**) Representative single-molecule fluorescence image of HA-Halo-MC3R (B) and examples of single-molecule fluorescence traces (C) with photobleaching steps (red arrows). (**D**) Proportion of molecules with 1 to 4 bleaching steps. N = 1565 molecules from 10 movies from 3 biological replicates. (**E** and **F**) Representative single-molecule fluorescence image of HA-Halo-MRAP2 (E) and examples of single-molecule fluorescence traces (F) with photobleaching steps (red arrows). (**G**) Quantification of molecules with 1 to 4 bleaching steps. N = 1333 molecules from 10 movies from 3 biological replicates. (**H**) Cells transfected with FLAG-CLIP-MRAP2 only, showing negligible background fluorescence. Representative image of 3 biological replicates. (**I** and **J**) Representative two-color SiMPull images of HA-Halo-MC3R and FLAG-CLIP-MRAP2 with colocalized spots circled in yellow (I) and photobleaching step analysis from colocalized spots (J). N=926 molecules from 10 movies from 3 biological replicates. Scale bar, 10 μm.

Although NanoBiT assays can indicate proximity between proteins, these assays do not measure interactions with single complex precision and cannot accurately measure stoichiometry. We therefore used the single-molecule pull-down (SiMPull) technique, which has previously been used to assess heteromeric GPCR complexes (26) (Fig. 2A). We generated MC3R and MRAP2 constructs with N-terminal hemagglutinin (HA) or FLAG epitopes followed by a SNAP, Halo or CLIP tag amenable to labelling with organic dyes (fig. S2A-B and S3A, and table S2). Using these constructs, we demonstrated that MC3R maintained receptor function (fig. S2C-D) and that MC3R and MRAP2 colocalized in cells when transiently transfected (fig. S3B), and confirmed that MRAP2 enhanced signaling by MC4R (fig. S3C). We first used SiMPull assays to determine the expression and stoichiometry of MC3R homomers. Cells were transfected with HA-Halo-MC3R, labelled with membrane impermeable CA-Sulfo646 (27), and lysed, then receptors were immobilized by binding of the HA tag to antibody-coated slides and single molecules were imaged by total internal reflection fluorescence microscopy. The majority of molecules showed single bleaching steps (~83%), whereas approximately 15% had two steps per molecule (Fig. 2B-D), indicating that most MC3R was monomeric at the cell surface. In the absence of HA antibodies, very few molecules (6 molecules across 5 images) were observed (fig. S4A). We also examined MRAP2 stoichiometry by SiMPull assays because MRAP2 has been described to form homodimers or higher order oligomers (2, 28, 29). We first verified that the dimeric GPCR mGluR2 produced single molecule with two photobleaching steps (30) (fig. S4B-D). In cells expressing HA-Halo-MRAP2 and labelled with CA-Sulfo646, MRAP2 showed single bleaching steps in ~68% of molecules, whereas ~28% had two bleaching steps, indicating some dimer formation may occur. A small number of molecules (<5%) had three or four bleaching steps corresponding to higher-order oligomers (Fig. 2E-G). Therefore, MRAP2 primarily forms stable monomers or dimers when expressed alone.

To assess MC3R and MRAP2 heteromers, HA-Halo-MC3R and FLAG-CLIP-MRAP2 expressed in HEK293 cells were labelled with CA-Sulfo646 and BC-DY547 fluorophores, respectively, prior to lysis and receptor immobilization followed by single-molecule imaging. In the absence of MC3R, there were negligible single molecules observed (13 molecules across 5 images) (Fig. 2H). In lysates from co-transfected cells, MRAP2 co-localization was seen for almost 30% of MC3R spots (fig. S4E-G). Photobleaching step analysis showed 1-step each for MC3R and MRAP2 in ~74% of co-localized spots, whereas some 2- and 3-step bleaching was observed for MRAP2 (Fig. 2I-J). Less than 5% of spots showed two MC3R and two MRAP2 bleaching steps. To verify these findings, the SiMPull experiments were repeated with the Halo and CLIP labels swapped such that cells were transfected with HA-Halo-MRAP2 and FLAG-CLIP-MC3R. FLAG-CLIP-MC3R expression alone produced few single molecules (24 molecules across 5 images) (fig. S4F). There were similar total numbers of co-localized spots (~32% of receptor spots) (fig. S4G). Bleaching step analysis of these spots showed 63% had one MC3R and one MRAP2 step, whereas 21% had two MRAP2 steps, ~7% had 3 steps for MRAP2, and ~7.5% had two steps each for MC3R and MRAP2 (fig. S4H-I). In contrast, MRAP2 did not pull-down or colocalize with SSTR3, a receptor that is not known to interact with MRAP2 and whose signaling is not enhanced by MRAP2 (fig. S5A-E). These studies indicate that MC3R is more likely to interact with MRAP2 in a 1:1 stoichiometry but can interact with more than one MRAP2 molecule.

### MRAP2 increases MC3R signaling

**Fig. 3:**
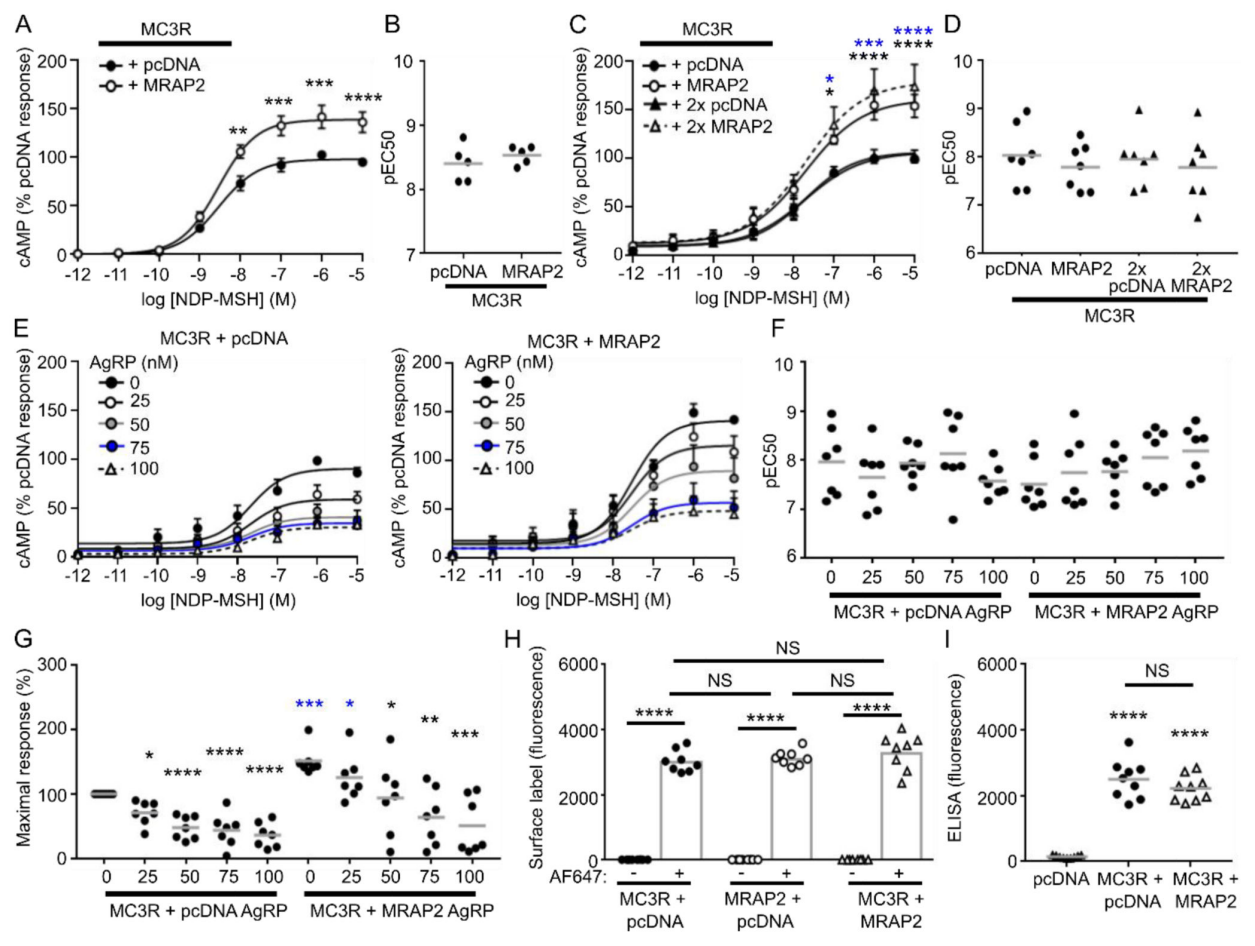
MRAP2 enhances MC3R signaling without affecting cell surface expression (**A**) MC3R-induced cAMP responses measured by GloSensor in cells transfected with pcDNA or MRAP2. AUC was measured and responses expressed relative to the pcDNA maximal response. N=5 biological replicates per group. (**B**) pEC_50_ values from (A). (**C**) MC3R-induced cAMP responses in cells cotransfected with 25 ng or 50 ng pcDNA and 25 ng or 50 ng MRAP2. N=7 biological replicates per group. AUC was measured and responses were expressed relative to the MC3R + 25 ng pcDNA maximal response. Statistical analyses show 25 ng pcDNA compared to 25 ng MRAP2 (black) or 50 ng MRAP2 (blue). (**D**) pEC_50_ values from (C). (**E**) Effect of the endogenous antagonist AgRP on MC3R-induced cAMP responses in cells cotransfected with pcDNA or MRAP2. The experiments were performed together but are presented side-by-side for easier visualization. N=7 biological replicates per group. Curves for lower concentrations are shown in fig. S6D. AUC was measured and responses were expressed relative to the pcDNA 0 nM AgRP maximal response. (**F** and **G**) pEC_50_ (F) and maximal responses (G) for (E). Statistical analyses show pcDNA vehicle compared to pcDNA AgRP (black), MRAP2 vehicle compared to MRAP2 AgRP (gray), pcDNA vehicle compared to MRAP2 responses (blue). (**H**) Surface labeling of cells transfected with SNAP-tagged MC3R, SNAP-MRAP2 or SNAP-MC3R with FLAG-CLIP-MRAP2 and labeled with SNAP-surface Alexa Fluor 647 (AF647). Fluorescence values were expressed relative to cells without the fluorescent label. N=8 biological replicates per group. (**I**) Cell surface expression of MC3R assessed by ELISA in cells transfected with FLAG-CLIP-MC3R and SNAP-MRAP2 or pcDNA. N=9 biological replicates per group. Data are expressed relative to pcDNA values. *p<0.05, **p<0.01, ***p<0.001, ****p<0.0001 by two-way ANOVA and Sidak’s for (A), (C) and (E); unpaired t-test for (B); one-way ANOVA with Dunnett’s test for (D) and compared 2:1 to 1:1 datasets; and one-way ANOVA with Sidak’s for (F) to (I). Data are presented as means ± SEM in (A), (C) and (E) and means in (B), (D) and (F) to (I). NS = non-significant.

Whether MRAP2 affects MC3R signaling has been unclear (2, 5, 19). Because our SiMPull data indicated that MRAP2 interacted with MC3R in a 1:1 stoichiometry, and there is no evidence that high concentrations of MRAP2 are required for its effects on MC3R, we performed our assays with equal concentrations of DNA. MC3R-induced increases in cAMP (as assessed by Glosensor assays) were observed in cells expressing equal concentrations of MC3R and MRAP2 (Fig. 3A-B). This effect was retained when as little as 25 ng of MC3R and MRAP2 was transfected, and transfecting 25ng of each plasmid compared to 500 ng improved fold-change responses (fig. S6A-C). Because the SiMPull data suggested MC3R could interact with MRAP2 dimers, cAMP signaling was also assessed after cotransfection of 25 ng MC3R and 50 ng MRAP2, which resulted in no significant differences compared to cells transfected with 25 ng MRAP2 and MC3R (Fig. 3C-D). Therefore, 25 ng of each plasmid were transfected in subsequent experiments to reduce overexpression artefacts. The endogenous antagonist AgRP still inhibited MC3R activity in the presence of MRAP2 at concentrations of 25-100 nM, and lower concentrations (0-10 nM) had no effect on MC3R responses (Fig. 3E-G, fig. S6D-F). AgRP reduced the maximal response of MC3R (25-100 nM) and MC3R with MRAP2 (50-100 nM) in a concentration-dependent manner, and 50 nM AgRP was sufficient to prevent the ability of MRAP2 to enhance MC3R responses (Fig. 3E-G). MRAP2 did not affect MC3R cell surface expression when assessed using cell impermeable SNAP-647 labeling and fluorescence quantification or ELISA (Fig. 3H-I). MRAP2 enhanced cAMP responses in mHypoE-39 cells that are of hypothalamic origin but do not endogenously express MC3R or MRAP2 at detectable levels (fig. S7A-B). Additionally, overexpression of untagged full-length native MRAP2 still enhanced signaling by MC4R and MC3R (fig. S7C-J), and untagged full-length native MC3R showed similar responses as HA-Halo tagged MC3R, demonstrating that the signal sequence in the SNAP, CLIP, or Halo constructs did not affect cAMP responses (fig. S7K-M).

### Identification of residues required for MC3R and MRAP2 interactions

**Fig. 4:**
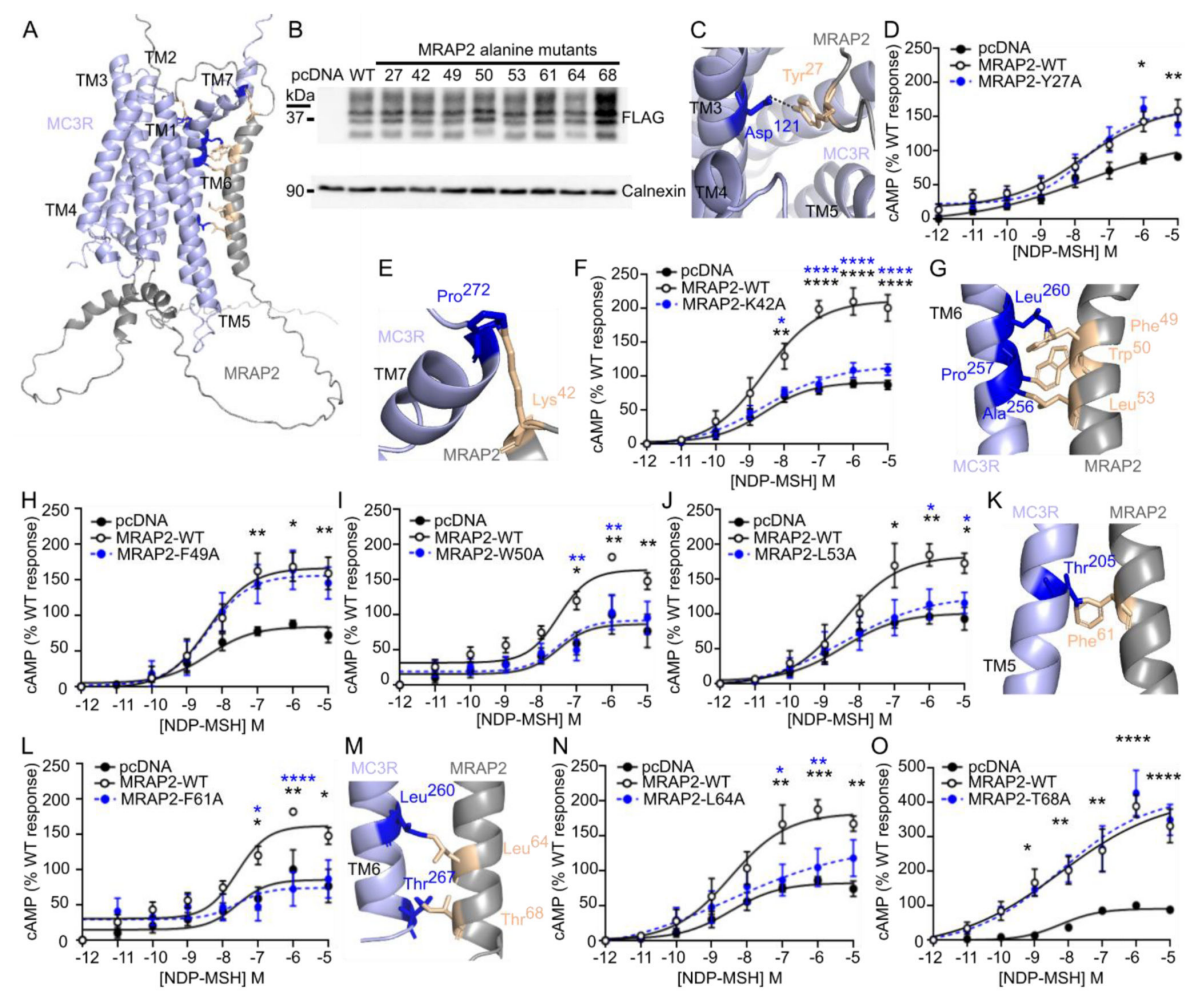
Prediction and assessment of MRAP2 residues that interact with MC3R (**A**) Predicted structural model of MC3R and MRAP2 in a 1-to-1 configuration with residues tested in cAMP assays highlighted in orange. (**B**) Western blot for FLAG-MRAP2 alanine mutants. Calnexin was used as a loading control. Representative of 4 biological replicates per group. Other blots are shown in fig. S11. (**C**) Predicted contacts between Tyr^27^ in MRAP2 and MC3R. The image shows the top of the structure with Tyr^27^ located close to the ligand-binding region of MC3R. (**D**) MC3R-induced cAMP responses in cells coexpressing MRAP2-Y27A. N=6 biological replicates per group. (**E** and **F**) Predicted contacts between Lys^42^ in MRAP2 and MC3R (E) and MC3R-induced cAMP responses in cells coexpressing MRAP2-K42A (F). N=7 biological replicates per group. (**G**) Predicted contacts between Phe^49^, Trp^50^, and Leu^53^ in MRAP2 and MC3R. (**H to J**) MC3R-induced cAMP responses in cells coexpressing MRAP2-F49A (H; N=7 biological replicates per group), W50A (I; N=5 biological replicates per group), or L53A (J; N=6 biological replicates per group). (**K** and **L**) Predicted contacts between Phe^61^ in MRAP2 and MC3R (K) and MC3R-induced cAMP responses in cells coexpressing MRAP2-F61A (L). N=5 biological replicates per group. (**M** to **O**) Predicted contacts between Leu^64^ and Thr^68^ in MRAP2 and MC3R (M) and MC3R-induced cAMP responses in cells coexpressing MRAP2-L64A (N; N=7 biological replicates per group) and MRAP2-T68A (O; N=4 biological replicates per group). All data were normalized to pcDNA maximal responses. Comparisons show MRAP2-WT compared to pcDNA (black) or MRAP2-WT compared to MRAP2 variant (blue). *p<0.05, **p<0.01, ***p<0.001, ****p<0.0001 by two-way ANOVA with Sidak’s or Dunnett’s multiple-comparisons test. pEC_50_ and E_max_ values are shown in table S5.

To understand how MRAP2 may interact with and facilitate MC3R signaling, we used AlphaFold2 to predict structural homology models. We first predicted the structure of MC3R and MRAP2 in a 1:1 stoichiometry because the SiMPull data indicated this was the most common form of the heterodimer. Of the five predicted models, one had multiple side chain collisions that could not be reduced with model refinement, and large unstructured regions, and therefore was not further assessed (fig. S8A and Movie S1). The other four models had a high confidence threshold and predicted that MRAP2 interacts with TM5-TM6 of MC3R (Movie S2), regions that have an important role in receptor activation and G protein coupling to MC3R (31). The models predicted that MRAP2 may insert within the membrane in two orientations (an extracellular N-terminus in two models and intracellular in the other models), consistent with previous studies (28, 32) (fig. S8B). The model ranked with the highest confidence (Model 1) contained more structured regions than the other models, including a loop close to the ligand-binding pocket of MC3R and a helical structure in the juxtamembrane G protein-binding region (Fig. 4A), similar to that observed in the MC2R-MRAP1 cryo-EM model (33).

Models 1, 2, 3, and 4 were assessed to determine all possible contacts between MRAP2 and MC3R, which identified twenty-two possible interactions observed in at least one model (table S3). We hypothesized that those residues identified in >3 models are more likely to be genuine contacts and therefore performed alanine mutagenesis of these residues in the FLAG-MRAP2 construct to determine whether they affected MC3R activity. We additionally assessed one residue (Thr^68^) located in the TM region close to these other residues that was predicted to form contacts in two models. All eight residues examined were highly conserved across mammalian species (fig. S9). Mutation of seven of these residues did not affect the total protein and cell surface expression of MRAP2 or MC3R (Fig. 4B, S10A-D, table S4). The T68A mutation significantly enhanced the total protein expression of MRAP2 (Fig. 4B, table S4, fig. S11) but did not affect the cell surface expression of either MRAP2 or MC3R (fig. S10A-D). Tyr^27^ is predicted to form contacts in all four models and lies in the ligand-binding region of MC3R in two models and the G protein docking region of two models (Fig. 4C, S8B). Mutation of Tyr^27^ to alanine did not affect MC3R-induced cAMP responses (Fig. 4D, table S5). Seven residues in the TM region (Lys^42^, Phe^49^, Trp^50^, Leu^53^, Phe^61^, Leu^64^, and Thr^68^) were predicted to form contacts with MC3R in multiple structural models (table S3). Mutation of Lys^42^, Trp^50^, Leu^53^, Phe^61^ and Leu^64^ to alanine reduced MC3R-induced responses such that they were indistinguishable from MC3R responses in the absence of MRAP2. Alanine mutagenesis of the other residues did not affect MC3R signaling (Fig. 4E-O, table S5).

**Fig. 5:**
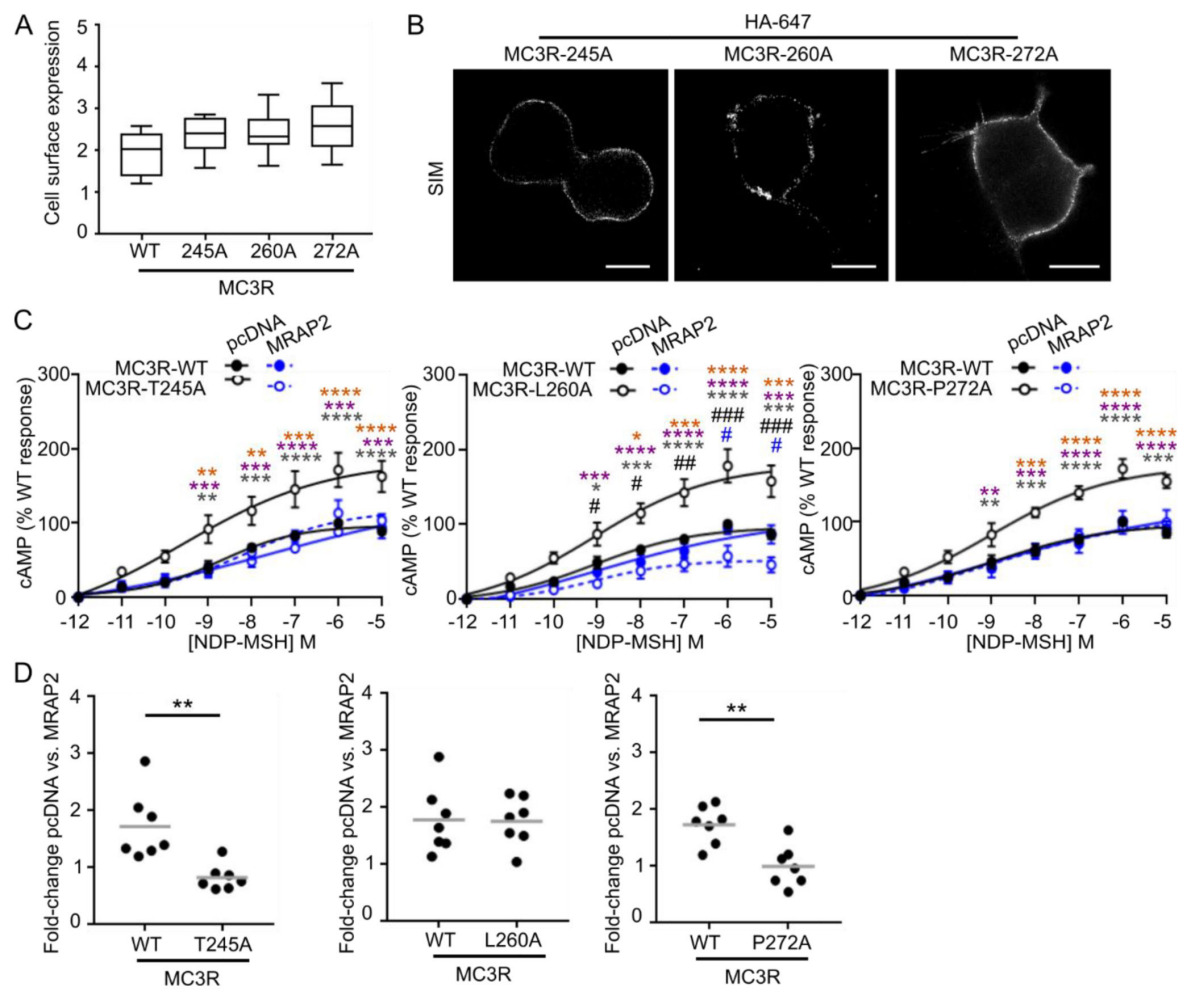
Assessment of MC3R residues that interact with MRAP2 (**A**) Fluorescent cell surface expression of WT or alanine mutants of MC3R. N=6 biological replicates per group. (**B**) SIM imaging of MC3R. Representative of 8-10 images from each of 3 biological replicates per group. Scale bar, 5 μm. (**C**) cAMP responses in cells cotransfected with MC3R-WT, MC3R-T245A, MC3R-T260A or MC3R-P272A with pcDNA or MRAP2. N=7 biological replicates per group. Comparison between MC3R-alanine variant with pcDNA and MC3R-WT with pcDNA (black hash) or MC3R-alanine variant with MRAP2 (blue hash). Asterisks compare MC3R-WT with MRAP2 to: MC3R-WT with pcDNA (orange), MC3R-alanine variant with pcDNA (purple), MC3R-alanine variant with MRAP2 (gray). Data are expressed relative to the maximal responses for MC3R-WT and pcDNA. (**D**) Maximum fold-change increase between pcDNA and MRAP2 transfected cells within each group from (C). *p<0.05, **p<0.01, ***p<0.001, ****p<0.0001 by two-way ANOVA with Sidak’s or Dunnett’s multiple-comparisons test in (C) and unpaired t-test in (D). pEC_50_ and E_max_ values are shown in table S6. ****p<0.0001, ***p<0.001, **p<0.01, *p<0.05.

To investigate the MC3R-MRAP2 interaction in further detail, we next mutated residues in MC3R that are predicted to interact with the five MRAP2 residues that affect MC3R-induced signaling (table S3). Alanine mutagenesis was performed on three MC3R residues (Thr^245^ in TM6, Leu^260^ in TM6, and Pro^272^ in TM7) that are highly conserved across mammalian species (fig. S9). Mutagenesis of the three residues to alanine did not affect MC3R cell surface expression (Fig. 5A-B), and the T245A and P272A MC3R variants did not show differences in agonist-induced responses in the absence of MRAP2. L260A reduced MC3R signaling and therefore, Leu^260^ may have a role in MC3R activation that is distinct from MRAP2-induced effects (Fig. 5C, table S6). The addition of MRAP2 did not further enhance MC3R-induced signaling by the T245A or P272A mutation above MRAP2-WT responses, suggesting that Thr^245^ and Pro^262^ may contribute to MC3R-MRAP2 interactions (Fig. 5C, table S6). MRAP2 potentiated MC3R-induced signaling by L260A, and although there was a significant difference between the absolute values for MC3R-WT and MC3R-L260A with MRAP2, the ability of MRAP2 to enhance MC3R-L260A responses was retained (Fig. 5C-D, table S6). Therefore, Leu^260^ may not have an important role in MC3R-MRAP2 interactions.

Because dimeric MRAP2 has been suggested to interact with MC3R, we also performed AlphaFold2 structural homology modelling with one MC3R and two MRAP2 residues. These models did not predict MC3R interactions with dimeric MRAP2 and instead predicted that the two MRAP2 residues may interact in two distinct sites on MC3R (fig. S8C and Movies S3X-SY. Because this result correlated with the SiMPull data that indicated binding of monomeric MRAP2 was preferential, we did not investigate these models in further detail.

### MRAP2 decreases MC3R internalization

**Fig. 6:**
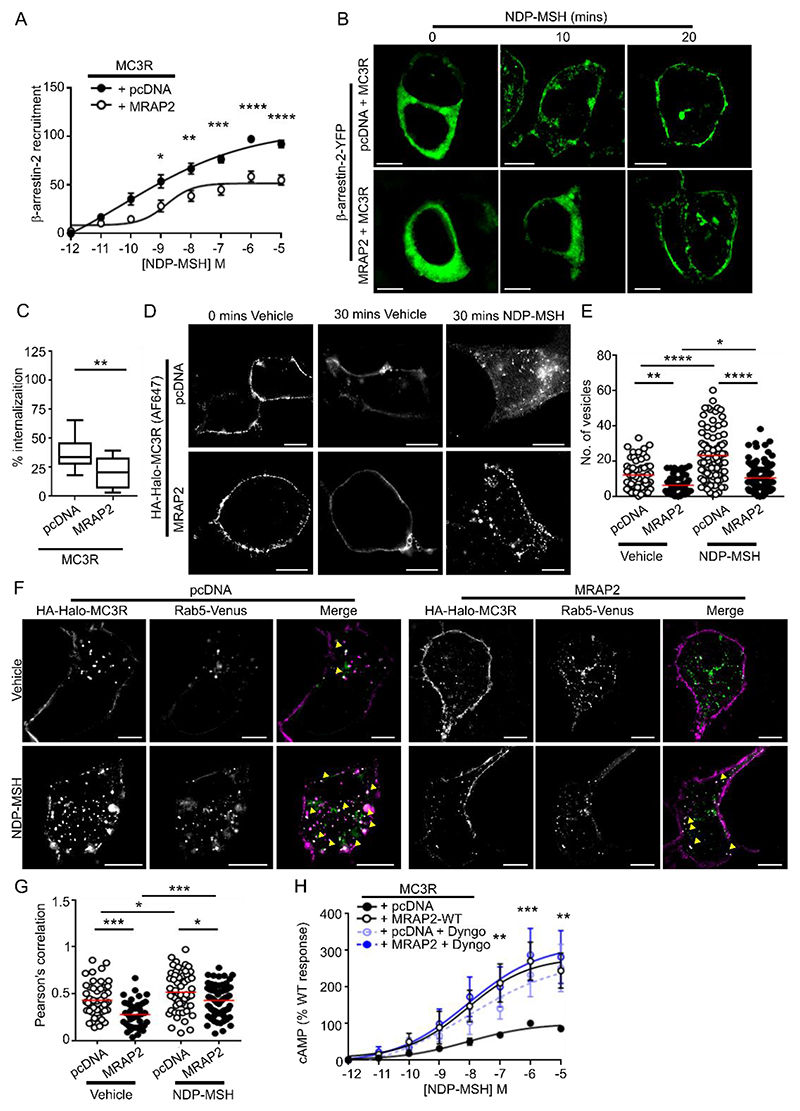
MRAP2 reduces β-arrestin recruitment and impairs receptor internalization (**A** and **B**) MC3R-induced membrane recruitment of β-arrestin-2 in cells cotransfected with pcDNA or MRAP2 as measured by BRET (A; N=8 biological replicates per group) and SIM (B; representative of 8-10 images for each of 4 biological replicates per group). Scale bar, 5 μm. BRET data are normalized to the maximal response for MC3R + pcDNA. (**C**) Percentage internalization of SNAP-MC3R following exposure to NDP-MSH for 30 minutes in cells cotransfected with pcDNA or MRAP2. Data are normalized to fluorescent values at 0 minutes. N=12 biological replicates per group. (**D**) Agonist-induced internalization as assessed by SIM imaging of BC-DY547-labeled MC3R in cells cotransfected with pcDNA or MRAP2. Scale bar, 5 μm. (**E**) Quantification of the number of internalized vesicles in cells exposed to vehicle or NDP-MSH for 30 minutes. 56-57 vehicle-treated cells and 91-93 agonist-treated cells from N=7 biological replicates per group. (**F**) SIM imaging of MC3R and Rab5 in cells expressing pcDNA or MRAP2. 41-60 cells from N=5 biological replicates per group. Scale, 5 μm. (**G**) Correlation assessed by Pearson’s coefficient between MC3R and Rab5 in SIM images. (**H**) MC3R-induced cAMP responses in cells cotransfected with pcDNA or MRAP2 and exposed to Dyngo (N=6 biological replicates per group). AUC was measured at each concentration and data are presented as normalized values relative to MC3R + pcDNA. *p<0.05, **p<0.01, ***p<0.001, ****p<0.0001 by two-way ANOVA with Sidak’s or Dunnett’s multiple-comparisons test in (A) and (H); one-way ANOVA with Sidak’s test in (E) and (G); and unpaired t-test in (C).

MRAP2 enhances GPCR signaling by impairing β-arrestin recruitment and consequently reducing receptor internalization (21, 23, 24). To determine whether MRAP2 uses a similar mechanism to enhance MC3R signaling, bystander BRET assays were performed measuring proximity between Nluc-tagged β-arrestin-2 and Venus-tagged Kras, a marker of the plasma membrane, in the presence of MC3R. BRET was enhanced in a concentration-dependent manner under all transfection conditions, although responses were significantly reduced in MRAP2-transfected cells when compared to control cells (Fig. 6A), suggesting that MRAP2 impairs MC3R-mediated β-arrestin-2 recruitment. SIM imaging of β-arrestin-2-YFP showed that under basal conditions, β-arrestin-2 was distributed across the cytoplasm in cells expressing MRAP2 or pcDNA control (Fig. 6B). In the presence of agonist, β-arrestin-2 was rapidly recruited to the plasma membrane in control cells. In contrast, in cells expressing MRAP2, β-arrestin-2 initially formed punctate structures and was recruited to the plasma membrane only after 20 minutes exposure to agonist (Fig. 6B).

Because MRAP2 impaired MC3R-induced β-arrestin-2 recruitment to the plasma membrane, we hypothesized that MRAP2 reduced receptor internalization. In cells expressing CLIP-MC3R and labeled with cell impermeable BC-DY547, ligand exposure reduced the surface labeling of MC3R, consistent with agonist-induced internalization of the receptor, which was attenuated by MRAP2 expression (Fig. 6C). SIM imaging of cells expressing HA-HALO-MC3R and incubated with an HA antibody revealed MC3R endocytosis in control and MRAP2-expressing cells exposed to the ligand NDP-MSH, although there appeared to be more internalized receptor in cells expressing MC3R without MRAP2 (Fig. 6D). MRAP2-expressing cells had fewer vesicles when treated with either vehicle or NDP-MSH when compared to cells expressing empty vector, indicating that both constitutive and agonist-driven MC3R internalization was reduced by MRAP2 (Fig. 6E). Consistent with the reduction in internalization, there was significantly less colocalization between the early endosome marker Rab5 and MC3R in MRAP2-expressing cells as assessed by SIM (Fig. 6F-G) in both vehicle- and agonist-exposed cells. Thus, MRAP2 impairs both constitutive and agonist-driven MC3R internalization.

These data suggest that MRAP2 may enhance MC3R signaling by reducing receptor internalization and thus increasing receptor retention at the cell surface. To assess whether blocking MC3R internalization results in an increase in receptor signaling, cells were pre-treated with Dyngo-4a, which impairs clathrin-mediated endocytosis (fig. S12). Impairment of internalization enhanced MC3R-induced cAMP responses as assessed by cAMP Glosensor assays in vector-transfected cells, such that these were no longer different to MRAP2 responses (Fig. 6H). Pre-treatment of MRAP2 expressing cells with Dyngo-4a did not affect MC3R responses, suggesting that impaired receptor internalization is a mechanism by which MRAP2 enhances GPCR signaling.

**Fig. 7:**
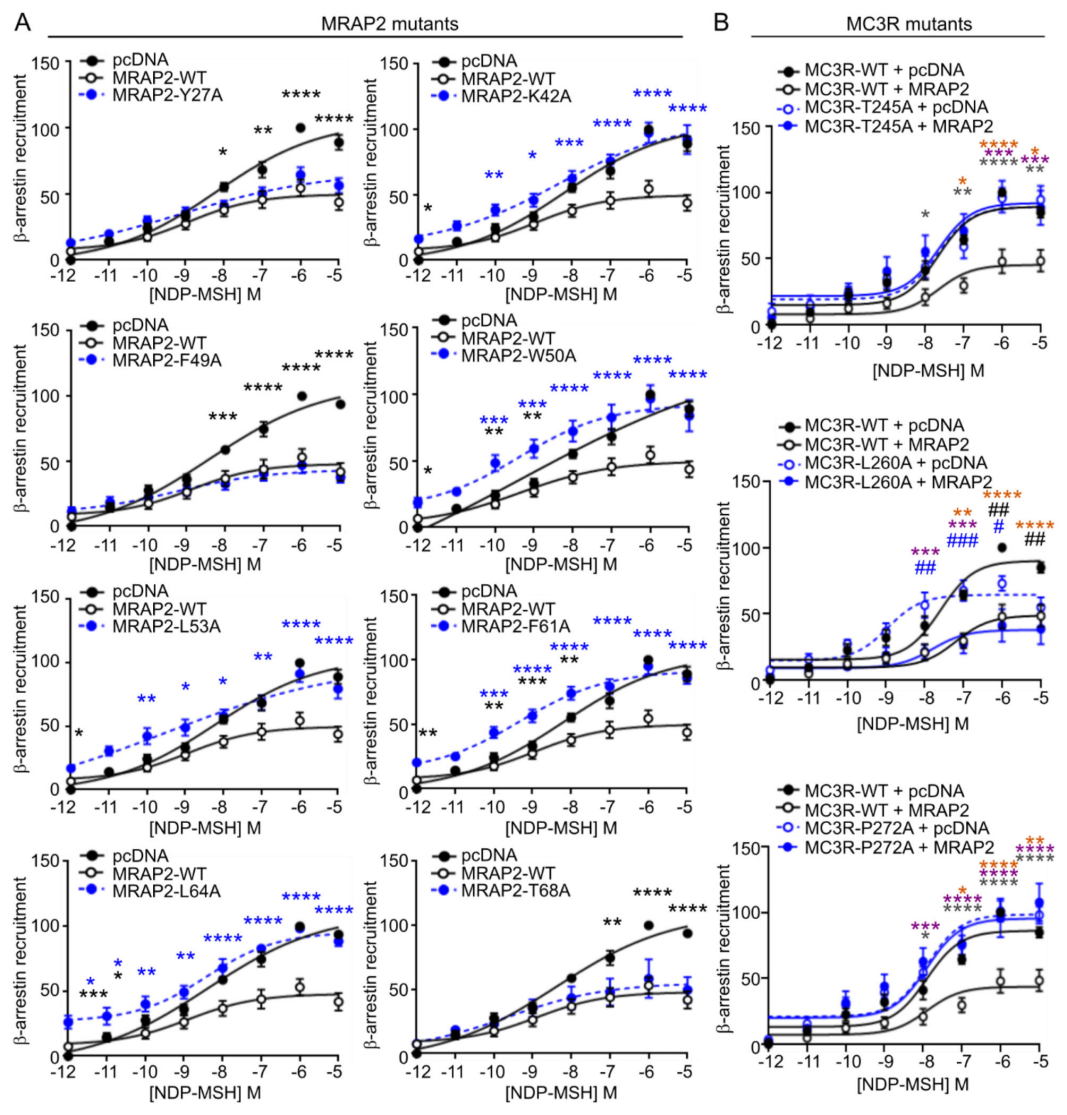
Effect of alanine mutations on β-arrestin signaling (**A**) MC3R-induced membrane recruitment of β-arrestin-2 as measured by BRET in cells transfected with MC3R and pcDNA, MRAP2-WT, or MRAP2 alanine mutants. N = 6-8 biological replicates per group. Statistical analyses were performed by two-way ANOVA with Sidak’s multiple-comparisons test. Comparisons show MRAP2 mutant compared to pcDNA (black asterisks) and MRAP2 mutant compared to MRAP2-WT (blue asterisks). Data are expressed relative to the maximal response for MC3R + pcDNA. (**B**) MC3R-induced membrane recruitment of β-arrestin-2 as measured by BRET in cells transfected with MC3R-WT, MC3R-T245A, MC3R-T260A and MC3R-P272A with pcDNA or MRAP2. N = 7 biological replicates per group. Data were expressed relative to the maximal response for MC3R-WT + pcDNA. *p<0.05, **p<0.01, ***p<0.001, ****p<0.0001 by two-way ANOVA with Sidak’s multiple-comparisons test. Comparison between MC3R-alanine variant and pcDNA and MC3R-WT with pcDNA (black hash) or MC3R-alanine variant with MRAP2 (blue hash). Asterisks compare MC3R-WT with MRAP2 compared to: MC3R-WT with pcDNA (orange), MC3R-alanine variant with pcDNA (purple), MC3R-alanine variant with MRAP2 (gray). pEC_50_ and E_max_ values are shown in tables S7 and S8.

To determine whether the putative interacting residues identified between MRAP2 and MC3R also affected β-arrestin recruitment, BRET assays were performed, which showed that the Y27A, F49A and T68A mutations in MRAP2 that did not affect cAMP signaling similarly did not affect β-arrestin recruitment (Fig. 7A). In contrast, expression of MRAP2 with alanine substitutions at Lys^42^, Trp^50^, Leu^53^, Phe^61^ or Leu^64^, which reduced MRAP2-induced increases in cAMP signaling, enhanced β-arrestin recruitment relative to MRAP2-WT-expressing cells but without enhancing E_max_ when compared to vector-transfected cells. The E_max_ values were higher than MRAP2-WT maximal responses (Fig. 7A, table S7). Expression of MC3R with alanine substitutions at Thr^245^ and Pro^272^ also enhanced β-arrestin recruitment such that it did not significantly differ from that in vector-transfected cells (Fig. 7B). Coexpression of MRAP2 in MC3R-T245A or MC3R-P272A expressing cells did not affect β-arrestin recruitment. In contrast, expression of MC3R-L260A impaired β-arrestin recruitment in vector-transfected cells, whereas MRAP2 coexpression with MC3R-L260A reduced β-arrestin recruitment (Fig. 7B, table S8). Thus, reduced cAMP responses by alanine mutations in MRAP2 or MC3R may be due to enhanced β-arrestin recruitment, and Leu^260^ may not have a major role in MRAP2 function.

### Obesity-associated variants in MRAP2 impair MC3R function

**Fig. 8:**
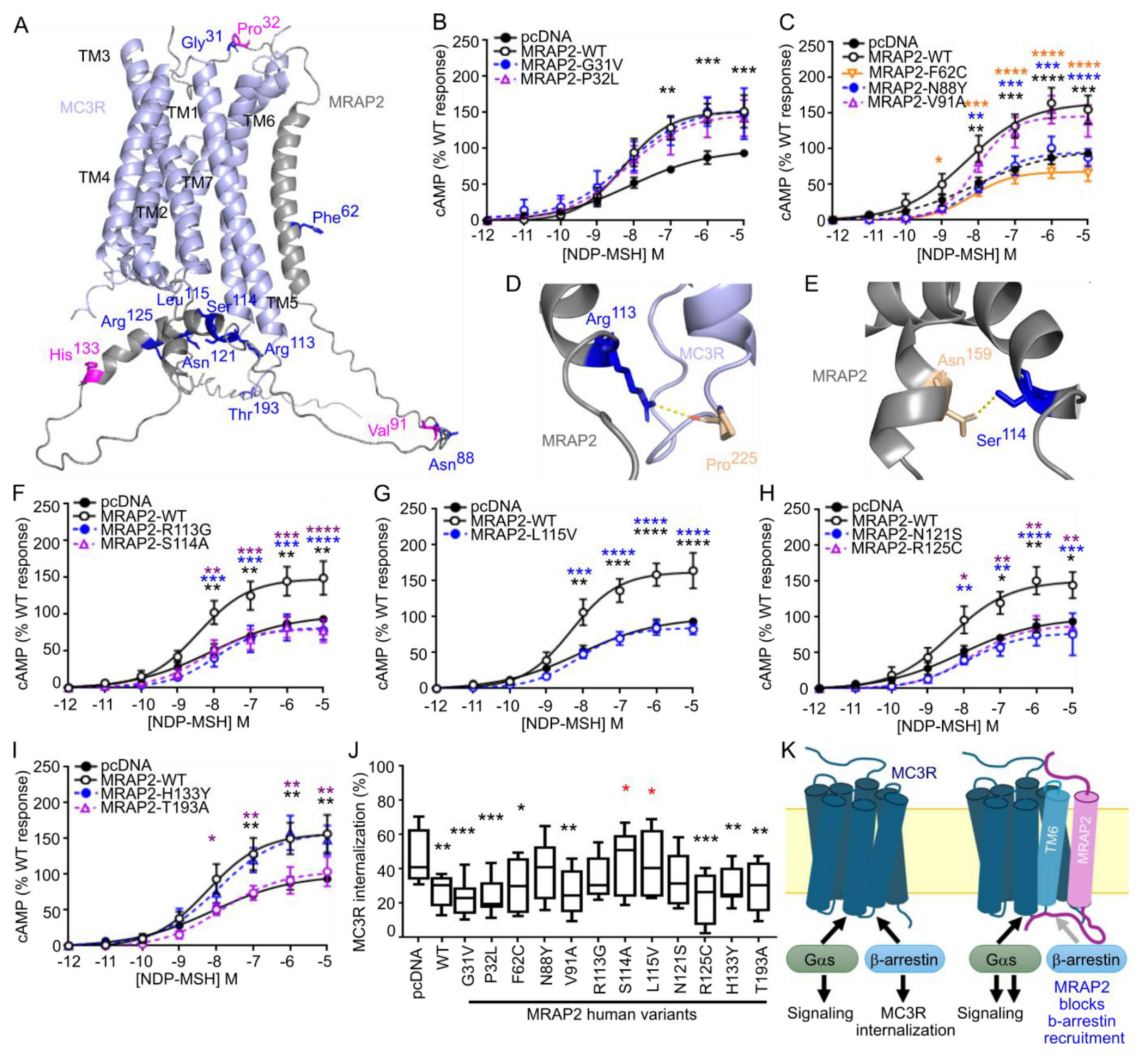
Effect of human MRAP2 variants on MC3R signaling and internalization (**A**) Predicted structural model of MC3R and MRAP2 in a 1-to-1 configuration with residues mutated in overweight or obese individuals. Residues highlighted in pink have been identified only in normal weight individuals. (**B**) MC3R-induced cAMP responses in cells cotransfected with MRAP2-G31V (N=7 biological replicates per group) or MRAP2-P32L (N=6 biological replicates per group). (**C**) MC3R-induced cAMP responses in cells cotransfected with MRAP2-F62C (N=7 biological replicates per group), MRAP2-N88Y (N=7 biological replicates per group) or MRAP2-V91A (N=5 biological replicates per group). (**D** and **E**) Predicted contacts between MC3R and MRAP2-R113 (D) or MRAP2-S114 (E). (**F**) MC3R-induced cAMP responses in cells cotransfected with MRAP2-R113G or MRAP2-S114A. (**G**) MC3R-induced cAMP responses in cells cotransfected with MRAP2-L115V. (**H**) MC3R-induced cAMP responses in cells cotransfected with MRAP2-N121S or MRAP2-R125C. (**I**) MC3R-induced cAMP responses in cells cotransfected with MRAP2-H133Y or MRP2-T193A. N=7 biological replicates per group for (F) to (H), N=6 biological replicates per group for (I). (**J**) MC3R-induced internalization in cells cotransfected with pcDNA, MRAP2 WT or an MRAP2 variant. N=10 biological replicates per group. *p<0.05, **p<0.01, ***p<0.001, ****p<0.0001 by two-way ANOVA with Sidak’s or Dunnett’s multiple-comparisons test. Asterisks compare MRAP2 WT to pcDNA (black) or variants according to their labeling in blue, orange or purple in (A) to (I). In (J), asterisks compare variants to pcDNA in black and to WT in red. (**K**) Cartoon of the proposed model in which the MRAP2 transmembrane region interacts with TM6 of MC3R and the cytoplasmic region of MRAP2 blocks β-arrestin recruitment to enhance Gαs signaling.

*MRAP2* genetic variants are associated with obesity, hypertension and diabetes (5, 6). Because these variants may affect the function of other GPCRs with which MRAP2 associates, we examined MC3R function in HEK293 cells expressing twelve different MRAP2 human variants. The twelve MRAP2 variants were selected based on their predicted location (Fig. 8A) either in the N-terminal ligand-binding region (G31V and P32L), transmembrane domain (F62C), C-terminal unstructured region (N88Y and V91A) or C-terminal helical structure within the G protein binding region (R113G, S114A, L115V, N121S, R125C, H133Y, and T193A). The variants did not significantly affect MC3R expression at the plasma membrane (fig. S13A-B). The G31V and P32L variants were not predicted to affect interactions with MC3R in the AlphaFold2 models (table S9) and expression of these variants did not affect MC3R-mediated cAMP responses (Fig. 8B). Phe^62^ in MRAP2 forms backbone interactions with other residues within the MRAP2 transmembrane helix (table S9). Expression of the variant MRAP2-F62C significantly impaired MC3R-mediated cAMP responses such that they did not significantly differ from vector-transfected cells. Expression of MRAP2-N88Y also significantly reduced MC3R-mediated cAMP responses (Fig. 8C). The R113G and S114A variants were predicted to lose contacts with adjacent MC3R and MRAP2 residues, respectively (Fig. 8D-E, table S9), and expression of these variants significantly impaired MC3R-induced cAMP signaling, as did that of the neighbouring L115V variant (Fig. 8F-G). Similarly, expression of three other C-terminal variants (N121S, R125C, or T193A) also significantly reduced MC3R activity (Fig. 8H-I).

Quantification of fluorescent surface labelling of MC3R after NDP-MSH exposure showed that fluorescence was reduced in cells expressing WT-MRAP2 or a variant, consistent with agonist-induced internalization. Similar to expression of WT-MRAP2, expression of most variants significantly decreased MC3R internalization compared to that in vector-transfected cells (Fig. 8J). Cells expressing the MRAP2 variants S114A or L115V showed MC3R internalization levels that were significantly different to those in cells expressing WT MRAP2 and instead were similar to those in vector-transfected cells, indicating that these variants impaired the effects of MRAP2 on internalization (Fig. 8J). Cells expressing the MRAP2 variants N88S, R113G, or N121S had an intermediate level of MC3R internalization that did not significantly differ from that in vector- or WT MRAP2-expressing cells, indicating that these variants may partially impair the effect of MRAP2 on internalization.

## Discussion

We showed that MRAP2 interacted with MC3R to enhance signaling, thus expanding the repertoire of receptors that robustly interact with MRAP2. Although MRAP2 has been described to interact with >40 GPCRs, signaling data has not been provided for most receptors, and often the data is not replicable between different groups (2, 3, 5, 13). In contrast, our data provides multiple lines of evidence demonstrating that MRAP2 facilitated MC3R signaling. First, we showed that *MC3R* and *MRAP2* are coexpressed in human neurons that regulate energy homeostasis and food intake and that the proportion of neurons that coexpress *MC3R* and *MRAP2* is similar to that coexpressing *MC4R* and *MRAP2*, the protein products of which are widely accepted to interact (Fig. 1A, S1, table S1). Secondly, the two proteins interacted at the single-molecule level, and MRAP2 enhanced signaling at low levels of expression (Fig. 2I-J and 3A). Thirdly, disruption of putative interacting residues impaired MRAP2-mediated signaling (Fig. 4A-O, 5C-D, and 7A-B). Fourthly, MRAP2 used similar mechanisms as those described for other receptors to impair β-arrestin recruitment (Fig. 6A-B). Finally, human variants in the MRAP2 transmembrane domain and C-terminus implicated in receptor interactions impaired signaling and affected internalization (Fig. 8B-J). Therefore, we are confident that MC3R and MRAP2 form heterodimers that contribute to MC3R function.

Several groups have shown that MRAP2 may form dimers at the cell surface (2, 19, 29), which has led to the assumption that MRAP2 exists in a dimeric form at the membrane which is necessary for its function (7, 34). However, MRAP2 dimerization has been analyzed using the isolated protein and therefore the effect of cotransfected GPCRs and heterodimer stoichiometry on MRAP2 were not established. Our SiMPull experiments (Fig. 2E-G) showed that MRAP2 could form dimers at the cell surface, consistent with previous findings, although monomeric MRAP2 was more prevalent. Moreover, most complexes comprise one MC3R molecule interacting with monomeric MRAP2 in cells coexpressing MC3R and MRAP2. Consistent with this stoichiometry, our structural homology models similarly predicted binding by monomeric MRAP2, and published structures of MC2R and MRAP1 also have a 1:1 stoichiometry (33). It is possible that when GPCR expression is low, MRAP2 may form dimers at the membrane, but when coexpressed with GPCRs, MRAP2 may heterodimerize with GPCRs in a monomeric form. However, our SiMPull analyses demonstrated a sizable proportion of MRAP2 was in a monomeric form at the cell surface when cells expressed only HALO-MRAP2, and it is therefore likely that there are both monomers and dimers at the cell surface, although our choice of detergent could have impacted these quantities. Our cell surface labeling strategy with membrane-impermeable dyes would also not be able to detect MRAP2 inserted in a C-terminal out orientation, and therefore we cannot discount that these dimers may also form. Examination of additional complexes will be required to determine whether this 1:1 stoichiometry is important for other MRAP2-GPCR interactions, although our analysis of MC4R suggest a 1:1 ratio for MC4R-MRAP2 complexes (15). Because our data and those of a preprint that was published during revision of our manuscript (23) showed that overexpression of MRAP2 was unnecessary and that lower DNA concentrations may improve fold-change responses (fig. S6A-B) future studies should also assess the stoichiometry between MC4R and MRAP2. Additionally, we suggest that signaling should be assessed by at least two independent assays with a full concentration-response curve, rather than a single concentration of agonist (7), because we showed that the maximal response may not be affected by some MRAP2 mutations or that constitutive activity may be affected. MRAP2 has been previously shown to affect the constitutive activity of other GPCRs (21). Our findings may have implications for other GPCR accessory proteins such as MRAP1 and RAMPs. MC2R has been suggested to interact with MRAP1 dimers (35) and subsequently MRAP1 has been overexpressed to reflect this. However, cryo-EM studies do not provide evidence for MRAP1 dimerization (33) and therefore, it may be necessary to assess signaling in cells cotransfected with equal concentrations of MC2R and MRAP2. Similarly, variable ratios of RAMPs:GPCR (from 1:1 to 20:1 RAMP:GPCR (36–39)) have been used, although structural studies again indicate that RAMPs form heterodimers with GPCRs in a 1:1 stoichiometry (39, 40). It may be important to verify interactions between GPCRs and interacting proteins with multiple assays and establish stoichiometry before assessing signaling effects in future studies.

We identified five residues in MRAP2 that may contribute to receptor interactions and/or facilitate signaling. These residues are all located in the transmembrane helix, a region that is important for the potentiation of GHSR signaling (21) by MRAP2. The transmembrane region is also important for MRAP1 interactions with MC2R (41), and it is likely that there is a shared mechanism by which these accessory proteins facilitate GPCR signaling. Cryo-EM structures demonstrated that MRAP1 interacts with TM5 and TM6 of MC2R (33). Our homology models predict that MRAP2 interacts with TM5 or TM6 of MC3R, and alanine mutagenesis of residues within TM6 impaired the ability of MRAP2 to facilitate MC3R signaling (Figure 8K). MC3R conforms to class A G protein coupling mechanisms whereby outward movement of TM6 allows formation of a large cytoplasmic cavity between TM5-TM7 that can accommodate G protein binding (31). Similar activation mechanisms have been described for MC2R (33) and MC4R (42), and we propose that MRAP2 transmembrane domain (TMD) interactions with TM5-TM6 of GPCRs allows the receptor to adopt a structural conformation that is more readily activated and/or allows G proteins to couple more efficiently. Such facilitation of a ‘partially preactivated state’ that can be more readily activated has been described for the ability of RAMP2 to potentiate signaling by the parathyroid hormone type-1 receptor (PTH1R) (39). Interactions between TM4 and TM5 of PTH1R and the RAMP2 transmembrane domain are important for establishing this preactivated state.

As with other GPCRs (21, 23, 24), we also found that MRAP2 impaired β-arrestin recruitment to MC3R. AlphaFold2 models suggest that the intracellular MRAP2 cytoplasmic region may sterically block the β-arrestin binding site (Figure 8K and Movies S2), which is likely to involve the intracellular ends of TM5 and TM6 (43). However, such a mechanism would also be expected to impair G protein coupling, suggesting that the cytoplasmic α-helix may undergo conformational changes following receptor activation. Disruption of the five TMD residues of MRAP2 that likely form interactions with MC3R enhanced β-arrestin recruitment by increasing constitutive activation, providing additional support for such a mechanism. This finding suggested that WT-MRAP2 might reduce constitutive β-arrestin recruitment, perhaps by blocking the β-arrestin binding site. Mutations in MRAP2 that disrupted either receptor interactions or conformational changes that facilitate G protein activation therefore enhanced constitutive β-arrestin recruitment and reduced G protein activation. Reduced β-arrestin recruitment and the consequent impairment in receptor internalization could explain some of the effects of MRAP2 on GPCR activation. Indeed, blocking MC3R endocytosis using Dyngo-4a enhanced receptor signaling and AgRP inhibits MC3R activity at least partially by enhancing β-arrestin recruitment and promoting receptor endocytosis (44). However, although we showed multiple MRAP2 variants impaired MC3R cAMP signaling, not all affected receptor trafficking, and it is possible that other mechanisms exist that allow MRAP2 to promote receptor signaling.

More than 25 *MRAP2* variants are associated with obesity (5–7, 14, 45). Kinetic assays performed on cells transfected with equal concentrations of MRAP2 and MC4R show impairment of cAMP and/or IP_3_ signaling by the obesity-associated variants (15). Hyperglycaemia and hypertension have been reported to occur more commonly in individuals with *MRAP2* mutations than in those with *MC4R* mutations, which led to the suggestion that MRAP2 variants may affect signaling by other GPCRs (6). This suggestion was also based on the finding that not all MRAP2 variants impaired MC4R function. We showed that MC3R-induced cAMP signaling was impaired by 8 MRAP2 variants but not by three variants (P32L, V91A, and H133Y) found exclusively in individuals with normal weight (6). Similarly, the P32L, V91A, or H133Y MRAP2 variants do not affect MC4R signaling (15), indicating these residues are unlikely to occupy an important role in MRAP2 structure-function. Whether inhibition of MC3R contributes to any of the clinical findings identified in individuals with MRAP2 variants is unknown. Mice with *Mc3r* deficiency have a high ratio of fat-to-lean mass but are not markedly obese unless fed a high-fat diet, and heterozygous mice have normal weight (46–48). However, mice with both *Mc3r* and *Mc4r* deficiency have a greater body weight than *Mc4r*^*-/-*^ mice (46), suggesting MC3R can contribute to weight gain. In contrast, depletion of *Mc3r* from AgRP neurons causes an anorexia and starvation phenotype, consistent with the orexigenic role of MC3R in these neurons (49). In humans, rare inactivating *MC3R* variants have been associated with obesity but not consistently (50). Several functionally inactivating *MC3R* heterozygous mutations have been linked to reduced height in childhood and early adulthood and a later onset of puberty but normal weight (51). One homozygous individual had also been overweight/obese since childhood and had type 2 diabetes and hypertension (51). Therefore, further studies of individuals with MRAP2 or MC3R variants are required to better understand how inactivating variants contribute to disease.

We also showed that MRAP2 variants can affect pathways other than the cAMP pathway, which is consistent with our findings for MRAP2 effects on MC4R. Five variants located in the intracellular domain that impaired cAMP signaling also reduced internalization. Structural homology models suggest part of the MRAP2 C-terminus may form an α-helical structure that lies within the MC3R cleft to which G proteins and β-arrestin bind. Several of the variants (R113G, S114A, L115V, and N121S) that affected both signaling and trafficking are present in this structure and we hypothesize that these variants disrupt the ability of the MC3R to engage with G proteins, resulting in impaired signaling. It will be important to investigate whether MRAP2 variants affect multiple aspects of GPCR signaling, because inactivating mutations in *MC4R* that contribute to obesity may not affect canonical signaling but can affect internalization, homodimerization or other G protein pathways (52). We showed that four of the MRAP2 variants located in the intracellular α-helix (R113G, S114A, L115V, and N121S) also impaired MC4R internalization (15), indicating there may be a shared mechanism for MRAP2 effects on GPCR activation. Further investigation of MRAP2 variants with other GPCRs could help determine whether this is a global mechanism or a melanocortin receptor-specific effect.

There are some limitations to the experiments presented in this manuscript. Data were primarily obtained from transfected HEK293 cells, which are commonly used in GPCR research, but do not endogenously express MC3R or MRAP2. Unfortunately, there are few cell lines derived from hypothalamic neurons, many of which are not models of NPY/AgRP neurons. In the mHypo-E39 hypothalamic cell line, overexpression of MC3R and MRAP2 in a 1:1 ratio increased MC3R signaling (fig. S7B). However, these cells do not endogenously express either MC3R or MRAP2 (fig. S7A), and therefore analysis of MRAP2-mediated signaling by endogenous proteins are still required. It is possible that human induced pluripotent stem cells (hiPSCs) may be useful for this purpose (53).

In summary, we showed that MRAP2 directly bound to MC3R enhanced Gα_S_-mediated signaling and impaired β-arrestin recruitment. Our mutagenesis studies and examination of human genetic variants demonstrated that the MRAP2 transmembrane domain and a putative C-terminal helix played an important role in facilitating MRAP2-mediated enhancement of MC3R activity, a mechanism that may apply to other GPCRs. Therapies that disrupt or enhance these sites could have important implications for treating disorders of food intake, including obesity and anorexia.

## Materials & Methods

### Plasmid constructs and compounds

A full list of plasmids and their sources can be found in table S2. For single molecule pull-down experiments, constructs were generated with an N-terminal signal peptide from rat mGluR2 (26), followed by affinity tags (HA or FLAG), self-labeling protein tags capable of conjugation to organic dyes (SNAP, CLIP, or Halo), and human MC3R and MRAP2. Cloning into the pRK5 vector was performed using reagents from Promega and oligonucleotides from Sigma to generate the following plasmids: ss-HA-Halo-MC3R, ss-HA-SNAP-MC3R, ss-FLAG-CLIP-MC3R, ss-HA-Halo-MRAP2, ss-HA-SNAP-MRAP2, ss-FLAG-CLIP-MRAP2, ss-HA-HALO-MC4R, ss-FLAG-CLIP-SSTR3. For ss-HA-Halo-GPCR and ss-HA-SNAP-GPCR, plasmids were constructed as follows: signal sequence (MVLLLILSVLLLKEDVRG), linker (SAQSTR), HA tag (YPYDVPDYA), linker (TRGSGS), Halo or SNAP tag, linker (RS), GPCR or MRAP2 gene. For ss-FLAG-CLIP-GPCR, plasmids were constructed as follows: signal sequence (MVLLLILSVLLLKEDVRG), linker (SAQSTRPV), FLAG tag (DYKDDDDK), linker (TRGS), CLIP tag, linker (RS), GPCR or MRAP2 gene. Full sequences of inserts for these plasmids (fig S14) have been uploaded to the University of Birmingham eData repository UBIRA (DOI: https://doi.org/10.25500/edata.bham.00001385). The MRAP2 variants were introduced into a MRAP2-3xFLAG plasmid by site-directed mutagenesis using the Quikchange Lightning Kit (Agilent Technologies) and oligonucleotides from Sigma. All plasmids were sequenced verified by Source Bioscience. NDP-MSH (Cambridge Bioscience) was used at 10 µM unless otherwise stated. Dyngo-4a (Abcam) was used at 30 µM to preincubate cells for 30 minutes prior to experiments. AgRP (Bio-Techne) was used between 0.01-100 nM to preincubate cells for ten minutes before reading plates.

### Cell culture and transfection

Adherent HEK293 cells were purchased from Agilent Technologies (AD-293, RRID:CVCL_9804) and mouse hypothalamic cells mHypoE-39 were purchased from Cedarlane Cellutions Biosystems Inc. (mHypo-N39, RRID:CVCL_D439). Both cell lines were maintained in DMEM-Glutamax media (Merck) with 10% calf serum (Merck) at 37ºC, 5% CO_2_. Cells were routinely screened to ensure they were mycoplasma-free using the TransDetect Luciferase Mycoplasma Detection kit (Generon). Expression constructs were transiently transfected into cells using Lipofectamine 2000 (LifeTechnologies) following the manufacturer’s instructions. To measure endogenous expression, total RNA was extracted from mHypoE-39 cells using RNeasy Mini Kits (Qiagen). cDNA synthesis was performed using the QuantiTect Reverse Transcription Kit (Qiagen). PCR was performed using oligonucleotides (Sigma). Mouse total RNA purchased from Takara bio (Catalog #636601) was used as a positive control. A total of 40 PCR cycles were used to ensure detection of genes with low expression.

### Transcript expression analysis

To assess the extent of co-expression of *MRAP2* with *MC3R* and *MC4R* separately we utilised HYPOMAP, a spatio-cellular atlas of the human hypothalamus (25). Log-normalized gene expression for *MRAP2* and *MC3R* was visualized in the spatial transcriptomics dataset. To highlight co-expression, spots which expressed both *MC3R* and *MRAP2* transcripts were highlighted. Using the single nucleus RNA-sequencing dataset, we calculated the percentage of neurons which expressed *MRAP2* across the whole hypothalamus dataset, as well as the percentage of *MC3R*-positive neurons that co-expressed *MRAP2* and the percentage of *MC4R*-positive neurons that co-expressed *MRAP2*. Co-expression was also measured on a cluster-by-cluster basis, at the highest resolution of clustering. To highlight co-expression in the snRNAseq dataset, cells which expressed MRAP2 and MC3R transcripts, or MRAP2 and MC4R transcripts were highlighted in the UMAP plots. Analysis and plots were performed using R and ggplot2.

### NanoBiT assays

NanoBiT assays were performed using methods adapted from a previous study (54). MRAP2 and MC3R were cloned into the LgBiT-C and SmBiT-C plasmids (purchased from Promega). HEK293 cells were seeded at 10,000 cells/well in 96-well plates and transfected the same day with 100ng (or as specified in the relevant figure legend) LgBiT and SmBiT plasmids. For competition studies with a single concentration, 100ng pcDNA or 100ng FLAG-MRAP2 were cotransfected. After 48 hours, media was changed to FluoroBrite DMEM phenol red-free media (Gibco) with 10% calf serum (FluoroBrite complete media) with 40 μL Nano-Glo substrate (Promega) and luminescence baseline signals were read on a Glomax (Promega) plate reader at 37°C. Data were normalized to luminescence values in the negative control sample (MC3R-SmC and LgC-Empty).

### Single molecule pull-down (SiMPull) assays

Cells were seeded in 12-well plates and transfected with 300 ng of Halo-tagged plasmids and 600ng of CLIP-tagged plasmids. After 24 hours, cells were washed with extracellular solution (comprising 135 mM NaCl (Sigma), 5.4 mM KCl (Sigma), 10 mM HEPES (Gibco), 2 mM CaCl_2_ (VWR Chemicals); 1 mM MgCl_2_ (Sigma), pH 7.4), and labelled with 2 μM of cell-membrane impermeable dyes (CLIP-surface 547 (BC-DY547, NEB) for FLAG-CLIP tagged plasmids or CA-sulfo646 for HA-Halo tagged plasmids) in extracellular solution for 45 min at 37°C. Cells were washed with extracellular solution and harvested in 1x Ca^2+^- and Mg^2+^-free PBS, and cell pellets were lysed in buffer (Tris pH8, NaCl, EDTA (all from Sigma)) containing 0.5% lauryl maltose neopentyl glycol/0.05% cholesteryl hemisuccinate (LMNG-CHS) (Anatrace) and protease inhibitor (Roche). Microflow chambers were prepared by passivating a glass coverslip and quartz slide with mPEG-SVA and biotinylated PEG (MW = 5000, 50:1 molar ratio, Laysan Bio) as previously described (26, 55). Before each experiment, a chamber was incubated with 0.2 mg/ml NeutrAvidin (Fisher Scientific UK) for 2 min, washed in T50 buffer (50 mM NaCl, 10 mM Tris), and incubated with 10 nM biotinylated HA antibody (ab26228, Abcam, RRID:AB_449023) in T50 buffer (50 mM NaCl, 10 mM Tris) for 30 minutes. Fresh cell lysates were mixed with dilution buffer (1:10 lysis working solution with extracellular solution) and added to the flow chamber until a suitable single molecule spot density was obtained. Chambers were washed with dilution buffer to remove unbound receptor, and single molecule movies were obtained as previously described (26) using a 100x objective (NA 1.49) on an inverted microscope (Olympus IX83) with total internal reflection (TIR) mode at 20 Hz with 50 ms exposure time with two sCMOS cameras (Hamamatsu ORCA-Flash4v3.0). Samples were excited with 561 nm and 640 nm lasers to excite BC-DY547 and CA-Sulfo-646, respectively. Single molecule movies were recorded sequentially at 640 nm, then at 561 nm until most molecules were bleached in the field. Images were analyzed using a custom-built LabVIEW program (56). Each movie was concatenated using MatLab (R2022a) and loaded on LabVIEW to visualize each channel for colocalized molecules. The fluorescence trace of each molecule was inspected manually and bleaching steps aligned. Data were plotted using GraphPad Prism.

### Three-dimensional modeling of MRAP2 and MC3R

Modeling of MC3R and MRAP2 was performed by AlphaFold2 using the ColabFold v1.5.2-patch in Google Co-laboratory (57) and visualized using Pymol. FASTA sequences were obtained from NCBI. Five models were predicted and ranked based on predicted local distance difference test (pLDDT). All models have been uploaded to the University of Birmingham eData repository UBIRA (DOI: https://doi.org/10.25500/edata.bham.00001385.

### Assessment of cell surface expression and internalization

To assess MC3R surface expression, cells were transfected with 100ng HA-HALO-MC3R and 100 ng pcDNA or FLAG-MRAP2 (WT or mutant). Cells were fixed 48 hours later in 4% paraformaldehyde (Fisher Scientific UK) in PBS and labelled with 1:1000 HA mouse monoclonal antibody (BioLegend Cat#901514, RRID:AB_2565336), then with 1:3000 Alexa Fluor 647 donkey anti-mouse secondary antibody (abcam Cat# ab181292, RRID:AB_3351687). Cells were washed, and fluorescence was read on a Glomax plate reader. Data were normalized to that observed in cells transfected with pcDNA, which was set as 1 and was not shown in graphs.

To assess MC3R internalization in the presence of MRAP2 mutants, HEK293 cells were seeded at 10,000 cells/well in 96-well plates and transfected the same day with 100 ng HA-SNAP-MC3R and 100 ng pcDNA or MRAP2 (WT or variants). Forty-eight hours later, cells were exposed to 10 μM NDP-MSH or vehicle for 30 minutes and labelled with 2 μM SNAP-surface-647 (NEB, Catalog# S91365).

### Western blot analysis

For MRAP2 mutant expression studies, either 3xFLAG-MRAP2-WT or 3xFLAG-MRAP2-mutants were transfected at 1 µg per well in a 6-well plate. Cells were lysed 48 hours later in NP40 buffer and Western blot analysis performed as previously described (58). Blots were blocked in 5% marvel/TBS-T and probed with 1:1000 mouse FLAG antibody (M2 antibody, Sigma-Aldrich, RRID:AB_262044) and 1:1000 rabbit calnexin antibody (Millipore, Cat# AB2301, RRID:AB_10948000). For untagged MRAP2 expression studies, either pcDNA, 3xFLAG-MRAP2-WT or pcDNA-MRAP2 (untagged) were transfected at 1 µg per well in a 6-well plate. Blots were probed with a rabbit polyclonal 1:1000 MRAP2 antibody (Proteintech, 17259-1-AP, RRID:AB_11182160) and 1:1000 calnexin antibody (Cat#AB2301). Blots were visualized using the Immuno-Star WesternC kit (BioRad) on a BioRad Chemidoc XRS+ system. Densitometry was performed using ImageJ (NIH), and protein signals were normalized to calnexin.

### Bioluminescence resonance energy transfer (BRET)

NanoBRET assays were performed using methods adapted from previous studies (59). HEK293 cells were seeded at 10,000 cells/well in 96-well plates and transfected the same day with 50 ng Nluc-Arr2, 500ng Venus-Kras, 100ng HA-Halo-MC3R and 100 ng pcDNA or FLAG-MRAP2. Forty-eight hours later, media was removed and replaced with Fluorobrite complete medium. Nano-Glo reagent was added at a 1:100 dilution and BRET measurements were recorded using a Promega GloMax microplate reader at donor wavelength 475-30 and acceptor wavelength 535-30 at 37°C. The BRET ratio (acceptor/donor) was calculated for each time point. After four baseline recordings were made, agonist was added at 8 minutes and recordings were made for an additional ~40 minutes. The average baseline value recorded prior to agonist stimulation was subtracted from the experimental BRET signal. All responses were normalized to the response in vehicle-treated cells to obtain the normalized BRET ratio. AUC was calculated in GraphPad Prism and these values were used to plot concentration-response curves with a 4-parameter sigmoidal fit.

### Glosensor cAMP assays

HEK293 cells were plated in 6-well plates and transfected with 200 ng pGloSensor-20F plasmid, and equal amounts of MC3R and MRAP2 (25-500 ng for transfection tests, and 25 ng for all other studies). Forty-eight hours later, cells were seeded in 96-well plates in FluoroBrite complete medium. Cells were incubated for at least 4 hours, and the media was changed to 100 µL of equilibration media consisting of Ca^2+^- and Mg^2+^-free HBSS containing 2% (v/v) dilution of the GloSensor cAMP Reagent stock solution (Promega). Cells were incubated for 2 h at 37°C. After reading basal luminescence on a Glomax plate reader for 8 min, agonist was added and plates were read for an additional 30 minutes. For FLAG-CLIP-SSTR3 studies, cells were preincubated with 10 μM forskolin for 5 minutes to elevate cAMP levels, and assays were performed as described for MC3R with somatostatin-14 (Sigma) added as the agonist. Data were plotted in GraphPad Prism. Area under the curve values were calculated and used to plot concentration-response curves with a 4-parameter sigmoidal fit.

### Structured illuminated microscopy (SIM)

Cells were plated on 24 mm coverslips (VWR) and transfected with 500ng of each plasmid at 36 hours before experiments. To measure cell surface expression, cells were fixed with 4% paraformaldehyde in PBS and incubated with 1:1000 anti-HA mouse monoclonal antibody (BioLegend Cat#901514, RRID:AB_2565336) or 1:1000 FLAG mouse monoclonal antibody (M2, Sigma-Aldrich, RRID:AB_262044), then to Alexa Fluor 647 goat anti-mouse (Cell Signaling Technology Cat# 4410, RRID:AB_1904023). For MC3R and MRAP2 colocalization studies, cells were incubated with HA rabbit primary antibody (ab26228, Abcam, RRID:AB_449023) and FLAG antibody, then with Alexa Fluor 647 goat anti-mouse (Cell Signaling Technology Cat# 4410, RRID:AB_1904023) and Alexa Fluor 488 goat anti-rabbit (Cell Signaling Technology Cat# 4412, RRID:AB_1904025). For studies with Rab5-Venus, cells were incubated with 1:1000 HA mouse monoclonal antibody (BioLegend Cat#901514, RRID:AB_2565336) with either vehicle or 10 μM NDP-MSH for 30 minutes. Cells were fixed, permeabilized and incubated with the Alexa Fluor 647 secondary antibody. Samples were imaged on a Nikon N-SIM system (Ti-2 stand, Cairn TwinCam with 2 × Hamamatsu Flash 4 sCMOS cameras, Nikon laser bed 488 and 647 nm excitation lasers, Nikon 100 × 1.49 NA TIRF Apo oil objective). SIM data was reconstructed using NIS-Elements (v. 5.21.03) slice reconstruction. Colocalization was measured and Pearson’s correlation coefficient was calculated using the ImageJ plugin JACoP. Vesicles were counted in ImageJ using protocols previously described (60). A manual threshold was set for each image to subtract background. Images were processed using ‘Process>Binary>Convert to mask’ and ‘Analyze particles’ in ImageJ. Images were manually inspected to ensure vesicles were captured by this method and observers were blinded to the experimental conditions.

### Statistical analysis

Statistical tests used for each experiment are indicated in the legends of each figure and the number of biological replicates (in which independent passages of cells were transfected on different days) denoted by N. For each biological replicate, 2-3 technical replicates were performed and the means used for combined data. Statistical analyses assumed no groups were paired (in other words, all groups were independent). Data were plotted and statistical analyses were performed in Graphpad Prism 7. Normality tests (Shapiro-Wilk or D’Agostino-Pearson) were performed on all datasets to determine whether parametric or non-parametric statistical tests were appropriate. A P value of <0.05 was considered statistically significant.

## Supplementary Material

Movie S1

Movie S2

Movie S3

Supplementary Materials

## Data Availability

Predicted structural models are available from the University of Birmingham eData repository UBIRA (DOI: https://doi.org/10.25500/edata.bham.00001385). All other data needed to evaluate the conclusions in the paper are present in the paper or in the Supplementary Materials. Plasmid constructs developed for this manuscript (table S2) will be provided by C.M.G. upon request.

## One-Sentence Summary

Signaling mediated by GPCR MC3R is boosted by a 1:1 association with the accessory protein MRAP2.

## Editor’s Summary

**Stimulating MC3R signaling with MRAP2**

The accessory protein MRAP2 enhances the activity of MC4R, a GPCR that regulates energy homeostasis. Whether it has a similar effect on the related MC3R has been controversial. Jamaluddin *et al*. showed that MRAP2 bound to MC3R in a 1:1 stoichiometry and enhanced its activity in a heterologous expression system. MRAP2 increased the ability of MC3R to stimulate the production of the second messenger cAMP by decreasing MC3R internalization, effects abolished by the mutation of residues in MRAP2 or MC3R involved in the interaction. *MRAP2* and *MC3R* mRNA expression overlapped in human hypothalamic neurons involved in energy homeostasis, and MRAP2 variants found in overweight and obese individuals did not enhance MC3R signaling. These results suggest that MRAP2 interacts with MC3R to stimulate its signaling.
