# Supplementary material for: The accessory protein MRAP2 directly interacts with melanocortin-3 receptor to enhance signaling
Source: Sci Signal. Author manuscript; Available in PMC 2025 Dec 23. (PMC7618510; doi:10.1126/scisignal.adu4315)
Supplement: Supplementary Materials [file EMS211715-supplement-Supplementary_Materials.pdf]

Supplementary Material

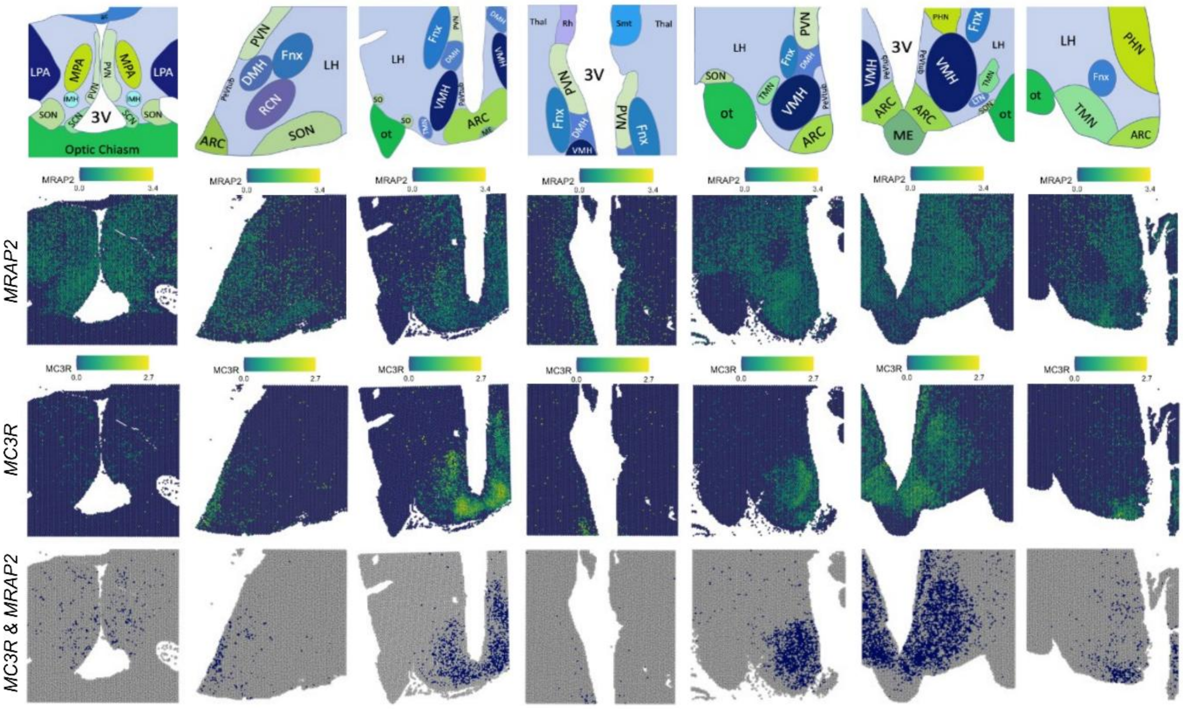

Fig. S1. Expression of *MRAP2* and *MC3R* in the human hypothalamus measured by spatial transcriptomics

Spatial transcriptomic data from (*25*) showing log-normalized expression of *MRAP2* and *MC3R* in the human hypothalamus. The cartoon shows the major regions of the hypothalamus (ARC, arcuate nucleus; DMH, dorsomedial hypothalamus; Fnx, fornix; LH, lateral hypothalamus, LTN, lateral tuberal nucleus; ME, medial eminence; MPA, medial preoptic area; PeVtub, periventricular nucleus; PHN, posterior hypothalamic nucleus; SCN, suprachiasmatic nucleus; SON, supraoptic nucleus; TMN, tuberomammillary nucleus, VMH, ventromedial nucleus of the hypothalamus), with areas in which *MRAP2* and *MC3R* are co-expressed shown at the bottom (indicated in blue).

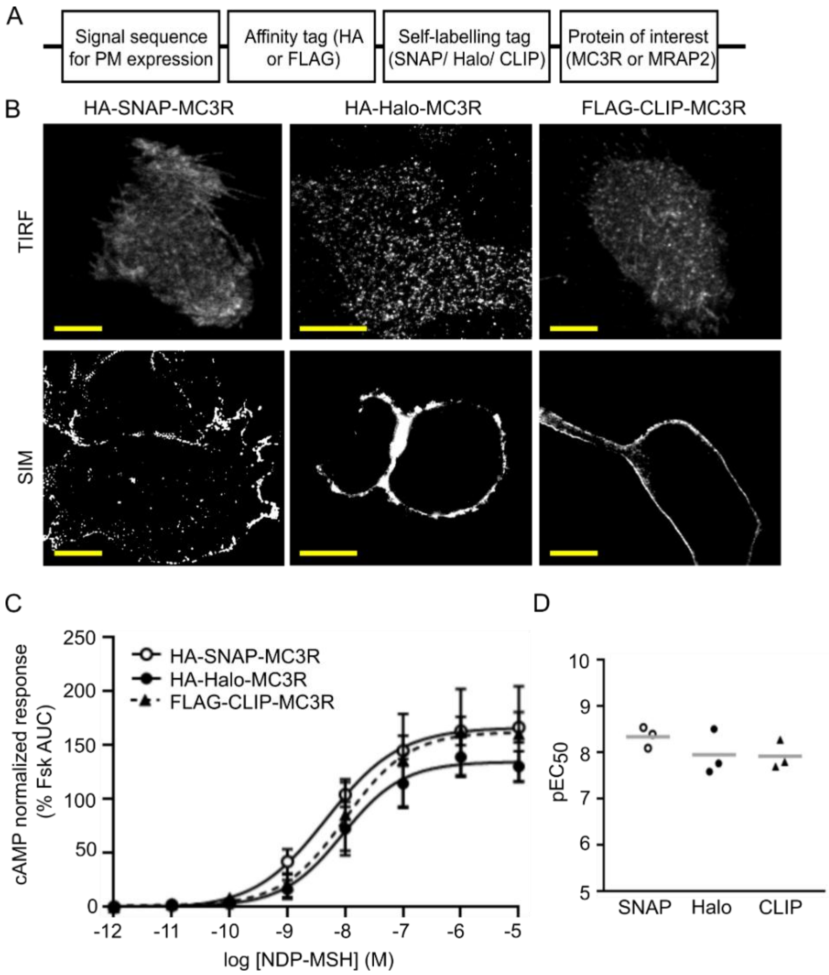

Fig. S2 **MC3R plasmids express, traffic and signal normally**

(**A**) The design of the MC3R and MRAP2 plasmids with a signal sequence, affinity tag (HA or FLAG), self-labeling tag (SNAP, Halo or CLIP) and the protein of interest. (**B**) TIRF and SIM imaging of the three MC3R constructs. Representative of 4 biological replicates per group. Scale bar, 5 µm. (**C**) MC3R-induced cAMP responses measured by Glosensor in cells transfected with one of the three MC3R plasmids (C) and pEC_{50} (D). AUC was used to generate a dose-response curve, which was expressed relative to basal responses. N=3 biological replicates per group. Statistical analyses were performed with two-way ANOVA with Sidak's multiple-comparisons test in (C) and with one-way ANOVA with Tukey's multiple-comparisons test in (D).

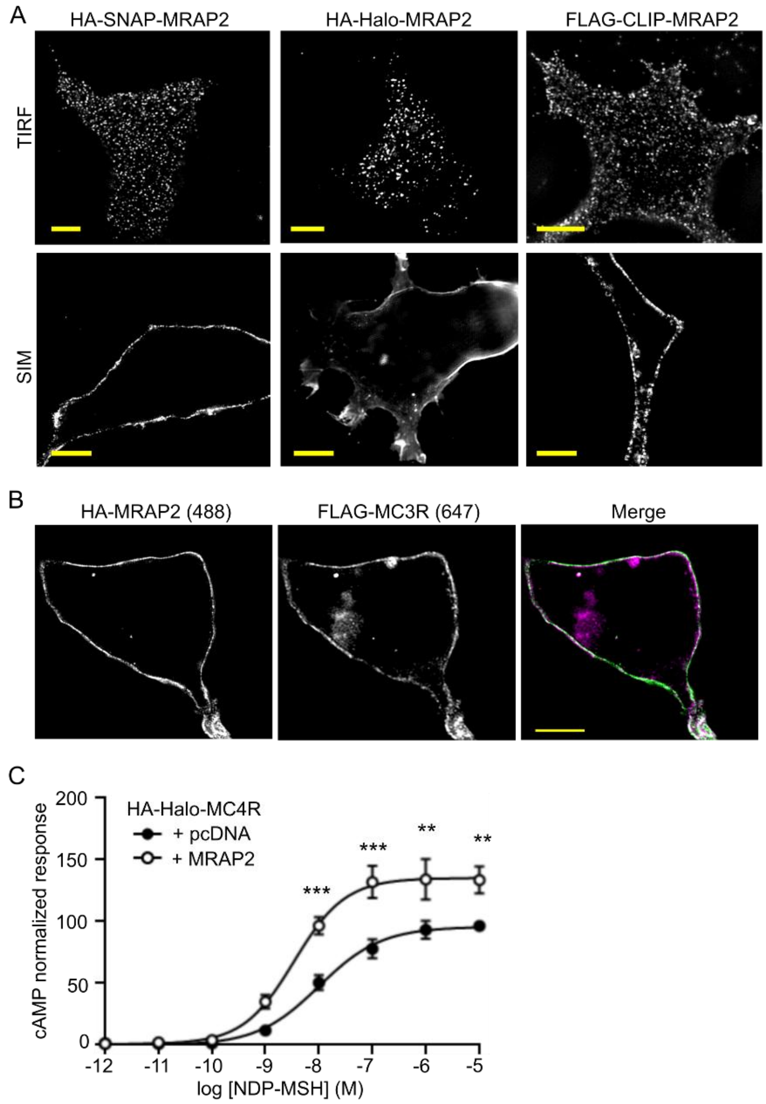

Fig. S3. MRAP2 plasmids express and signal normally
(**A**) TIRF and SIM imaging of the three MRAP2 constructs. Representative of 4 biological replicates per group. Scale bar, 5 µm. (**B**) SIM imaging of FLAG-CLIP-MC3R and Halo-HA-MRAP2. Representative of 4 biological replicates per group. Scale bar, 5 µm. (**C**) MC4R-induced cAMP responses measured by Glosensor in cells cotransfected with pcDNA or MRAP2. AUC was measured and expressed relative to the maximal response for pcDNA. N=4 biological replicates per group. **p<0.01, ***p<0.001 by two-way ANOVA with Sidak's multiple-comparisons test.

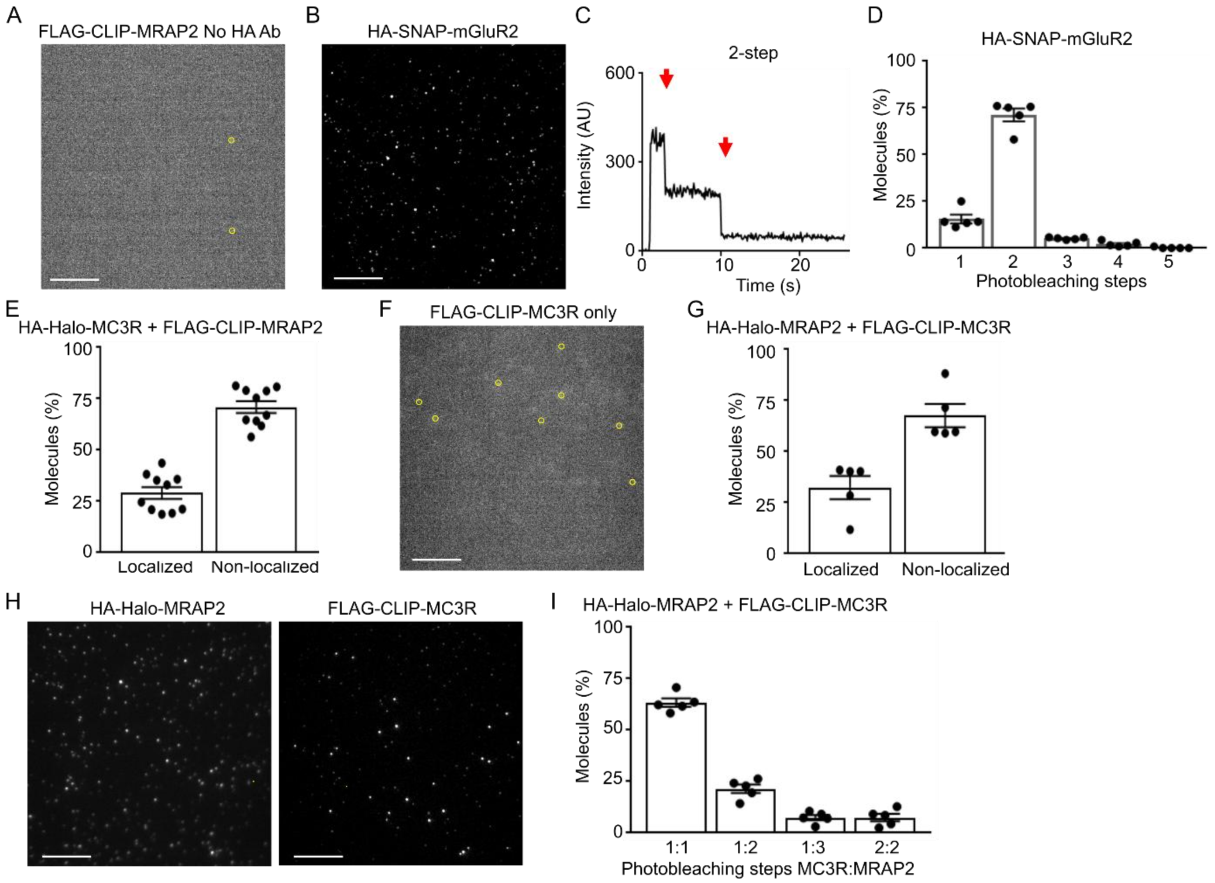

Fig. S4. MC3R and MRAP2 interact in a 1:1 stoichiometry

(**A**) Representative image showing little background fluorescence in the absence of HA antibodies. Background fluorescent spots are shown with yellow circles. Representative of 2 biological replicates per group. (**B** and **C**) Representative single-molecule fluorescence image of HA-SNAP-mGluR2 (B) with examples of single-molecule fluorescence traces (C) with photobleaching steps (red arrows). (**D**) Proportion of molecules with 1 to 5 bleaching steps. N=1425 molecules from 5 movies from 2 biological replicates. (**E**) Proportion of molecules in two-color SiMPull that are colocalized in cells transfected with HA-Halo-MC3R and FLAG-CLIP-MRAP2. N=3011 molecules from 10 movies from 3 biological replicates. (**F**) Cells transfected with FLAG-CLIP-MC3R only, showing negligible background fluorescence. Background fluorescent spots are shown with yellow circles. Representative of 3 biological replicates. (**G**) Proportion of molecules in two-color SiMPull that are colocalized in cells transfected with HA-Halo-MRAP2 and FLAG-CLIP-MC3R. N=1608 molecules from 5 movies from 3 biological replicates. (**H** and **I**) Representative two-color SiMPull images of HA-Halo-MRAP2 and FLAG-CLIP-MC3R (H) and photobleaching step analysis from colocalized spots (I). N=537 molecules from 5 movies from 3 biological replicates. Scale bar, 10 µm.

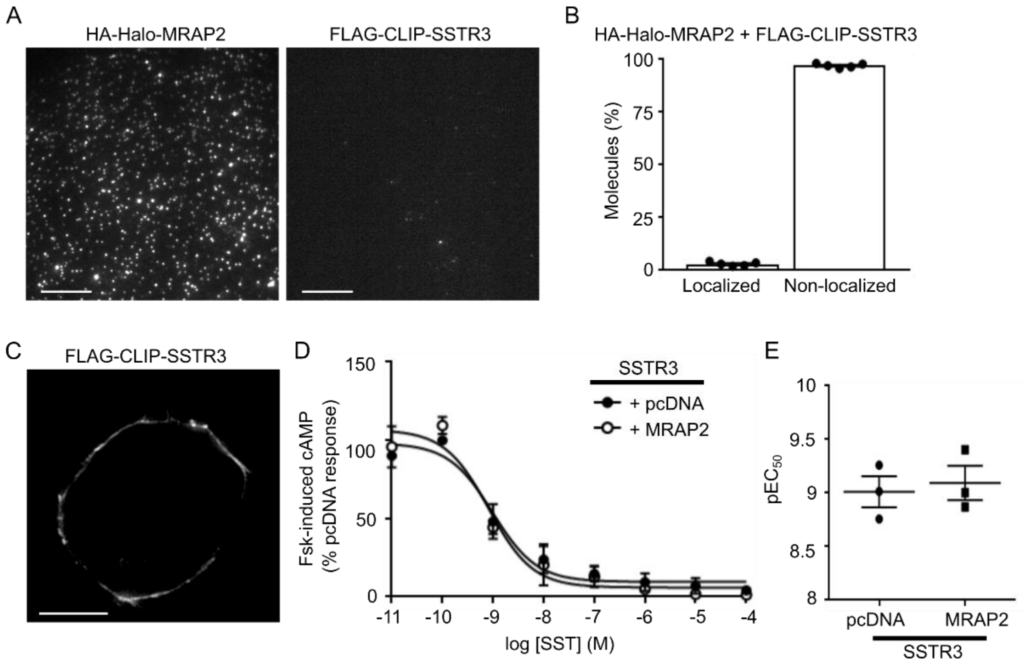

Fig. S5. MRAP2 does not interact with SSTR3

(**A** and **B**) Representative two-color SiMPull images of HA-Halo-MRAP2 and FLAG-CLIP-SSTR3 (A) and quantification of the proportion of molecules that are colocalized (B). N=1790 molecules from 5 movies from 3 biological replicates. Scale bar, 10 µm. (**C**) SIM image of FLAG-CLIP-SSTR3. Representative of 4 biological replicates. Scale bar, 5 µm. (**D**) Effect of FLAG-CLIP-SSTR3 on forskolin (Fsk)-induced cAMP production in cells cotransfected with pcDNA or MRAP2, with (**E**) pEC50 values. N=3 biological replicates per group. Statistical analyses comparing responses at each concentration of agonist were performed by two-way ANOVA with Tukey's multiple-comparisons test and those comparing pEC_{50} were performed by unpaired t-test.

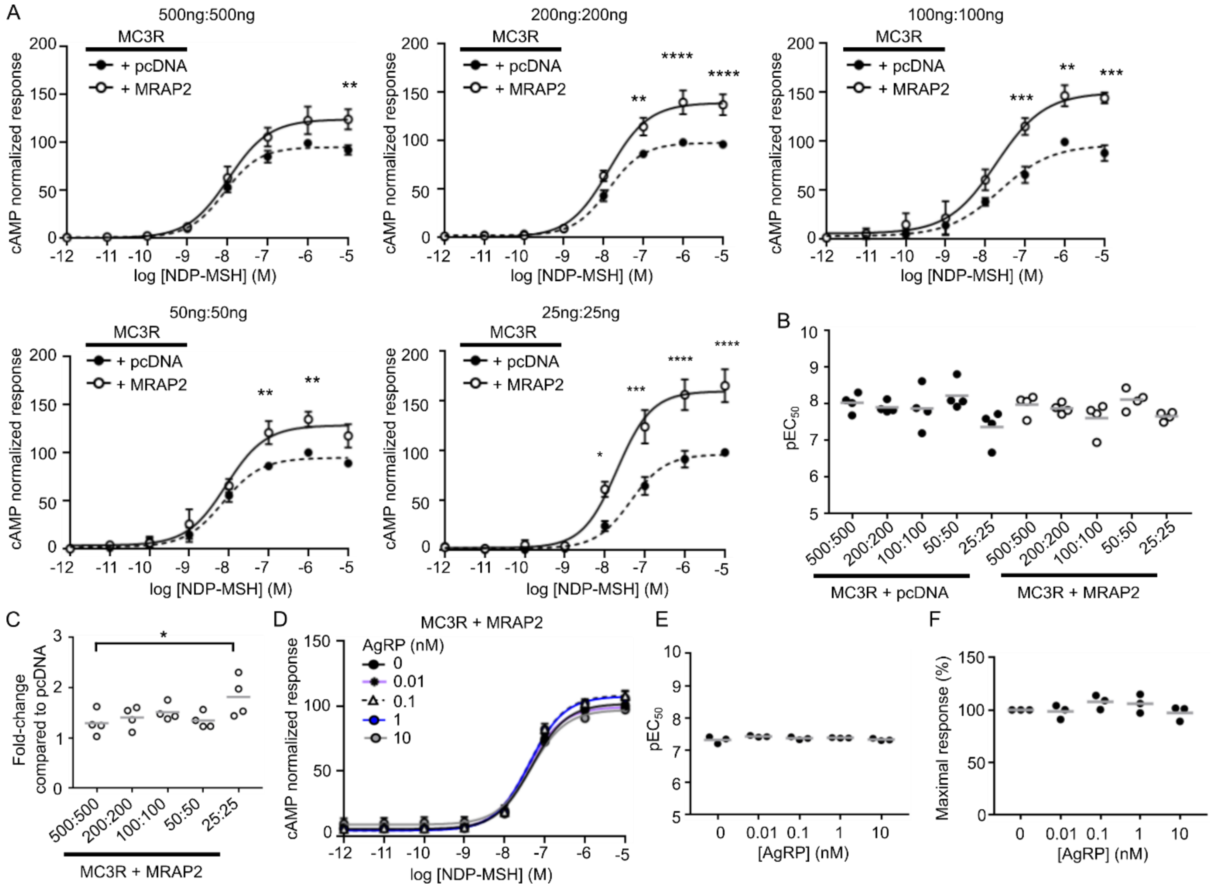

Fig. S6. MRAP2 enhances MC3R activity when equal DNA concentrations are transfected

(**A**) cAMP responses as measured by Glosensor in cells transfected with different total amounts of MC3R with pcDNA or MRAP2. The same DNA concentrations of MC3R and pcDNA or MRAP2 were transfected. AUC was measured and responses were expressed relative to the maximal response for pcDNA. N=4 biological replicates per group. Statistical analyses were performed by two-way ANOVA with Sidak's test. (**B**) pEC_{50} values from (A) compared using one-way ANOVA with Tukey's multiple comparisons test. (**C**) Maximal fold-change responses in MC3R-induced cAMP responses in cells transfected with pcDNA and MRAP2 at each DNA ratio. (**D**) Effect of 0-10 nM AgRP on MC3R-induced cAMP responses in cells transfected with MRAP2. N=3 biological replicates per group. (**E** and **F**) pEC_{50} values (E) and maximal responses (F) from (D). *p<0.05, **p<0.01, ***p<0.001, ****p<0.0001 by one-way ANOVA with Dunnett's multiple comparisons test in (C), (E), and (F).

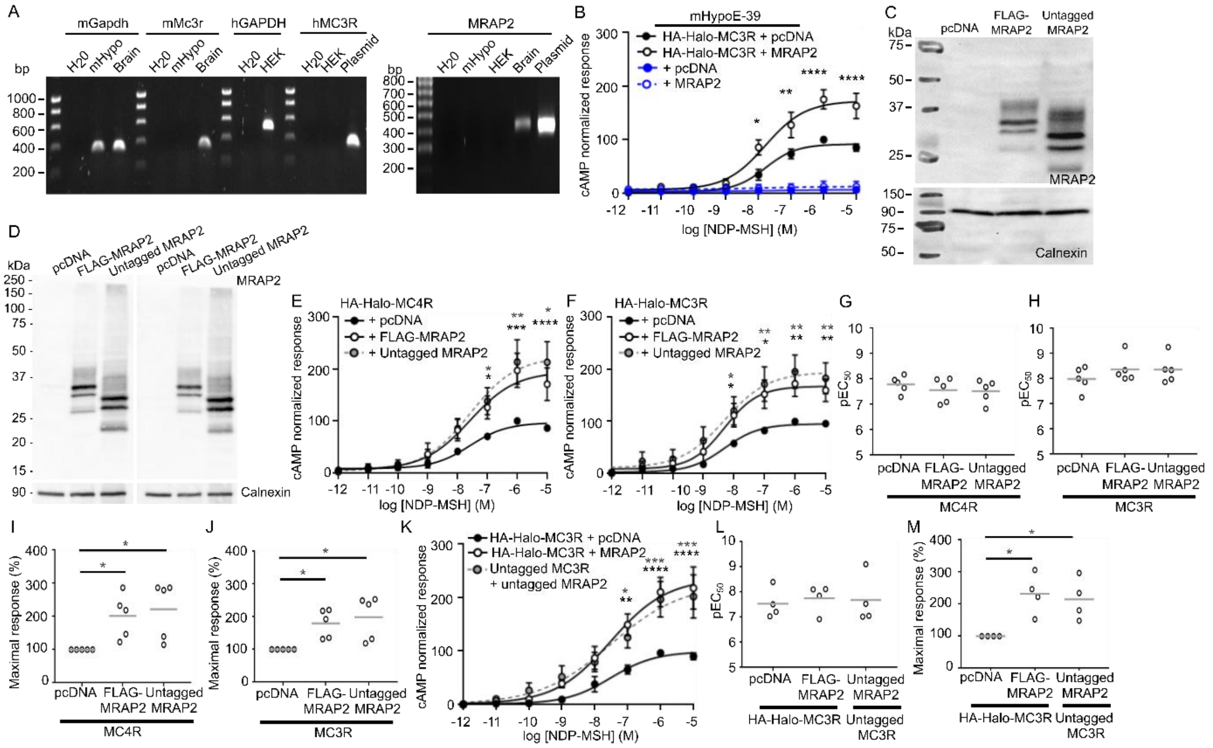

Fig. S7. Native MC3R and MRAP2 signal normally

(**A**) RT-PCR analysis of endogenous *Gapdh* (used as a control gene) and *Mc3r* or *Mrap2* in mHypoE-39 (mHypo) cells or mouse brain. In HEK293 cells, endogenous expression of *GAPDH*, *MC3R* and *MRAP2* were compared to a plasmid expressing the human *MRAP2* or *MC3R* gene. Representative of 3 biological replicates per group. (**B**) cAMP responses as measured by Glosensor in mHypoE-39 cells transfected with either pcDNA or MRAP2 (to detect endogenous responses) or HA-Halo-MC3R with either pcDNA or MRAP2. Statistical analyses compared pcDNA to MRAP2 responses in HA-Halo-MC3R transfected cells (black). N=6 biological replicates per group. (**C-D**) Western blots showing expression of pcDNA, FLAG-MRAP2 or untagged pcDNA-MRAP2 in HEK293 cells. Data shows lysates from 3 biological replicates per group. (**E** to **J**) cAMP responses in HEK293 cells transfected with either pcDNA, FLAG-MRAP2 or untagged pcDNA-MRAP2 and HA-Halo-MC4R (E) or HA-Halo-MC3R (F). pEC_{50} values for MC4R (G) and MC3R (H) with maximal responses for MC4R (I) and MC3R (J). N=5 biological replicates per group. (**K** to **M**) cAMP responses in HEK293 cells transfected with HA-Halo-MC3R or untagged pcDNA-MC3R and pcDNA or FLAG-MRAP2 (K)and pEC_{50} values (L) and maximal responses (M). N=4 biological replicates per group. In (E) and (F), statistical analyses compared receptor with pcDNA to FLAG-MRAP2 (black) and with pcDNA to untagged MRAP2 (gray). In (I), statistical analyses compared HA-Halo-MC3R with pcDNA to HA-Halo-MC3R with MRAP2 (black) and to untagged MC3R and MRAP2 (gray). Statistical analyses were performed by two-way ANOVA with Sidak's multiple comparisons test in (B), (E), (F), and (I) and one-way ANOVA with Dunnett's multiple comparisons test in (G), (H), and (J).

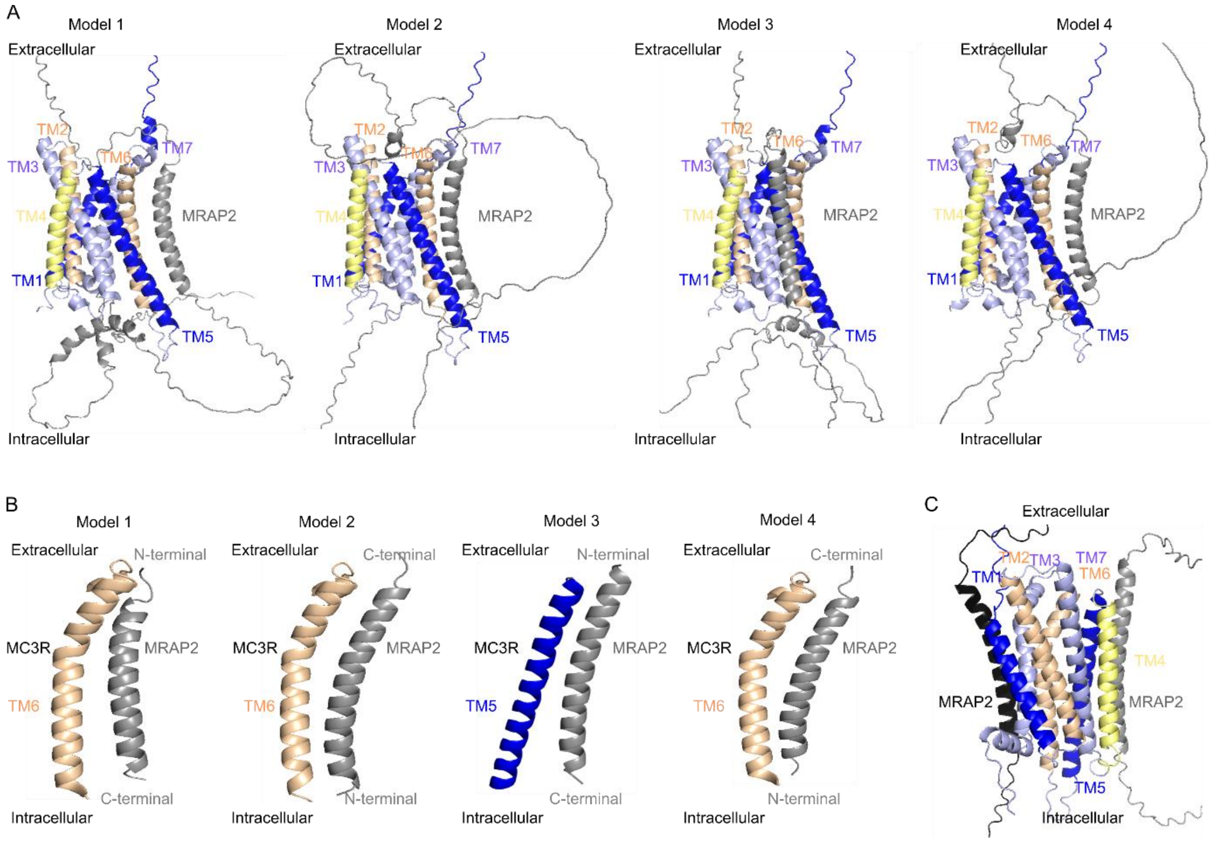

Fig. S8. AlphaFold2 models showing predicted interactions between MC3R and MRAP2

(**A**) Predicted structural models of MC3R and MRAP2 interactions. (**B**) The four models predicted that MRAP2 interacts with TM5-TM7, which are important for receptor activation and G protein coupling to MC3R. (**C**) Predicted structural model between MC3R and two MRAP2 proteins.

```
MRAP2 Human    ---------------------MSAQRLISNRTSQQSASNSDYTWEYEYYEIGPVSFEGLK       39
MRAP2 Mouse    -------------------MEMSAQRLASNRTSPQSPSNSDYTWEYEYYEIGPVSFEGLK       41
MRAP2 Rat      -------------------MEMSAQRLTSNRTSPQSPSNSDYTWEYEYYEIGPVSFEGLK       41
MRAP2 Cow      --MPTNLE-NLSVATGLEKVEMSAQRLISNRTAQQSASNSDYTWEYEYYEIGPVSFEGLK       57
MRAP2 Dog      MINPHFWDAEAEARATRTKVEMSAQRLISNRTSQPSAPNSDYTWEYEYYEIGPVSFEGLK       60
                                     ****** ****:  *  *********************

MRAP2 Human    AHKYSIVIGFWVGLAVFVIFMFFVLTLLTKTGAPHQDNAESSEKRFRMNSFVSDFGRPLE       99
MRAP2 Mouse    AHKYSIVIGFWVGLAVFVIFMFFVLTLLTKTGAPHQDNAESSERRFRMNSFVSDFGKPLE       101
MRAP2 Rat      AHKYSIVIGFWVGLAVFVIFMFFVLTLLTKTGAPHQDNAESSEKRFRMNSFVSDFGKPLE       101
MRAP2 Cow      AHKYSIVIGFWVGLAVFVIFMFFVLTLLTKTGAPHQDNAESSEKRFRMNSFVSDFGKPLE       117
MRAP2 Dog      AHKYSIVIGFWVGLAVFVIFMFFVLTLLTKTGAPHQDNAESSEKRFRMNSFVSDFGRPLE       120
               ******************************************:************:***

MRAP2 Human    PDKVFSRQGNEESRSLFHCYINEVERLDRAKACHQTTALDSDVQLQEAIRSSGQPEEELN       159
MRAP2 Mouse    SDKVFSRQGNEESRSLFHCYINEVEHLDRVKVCHQTTAIDSDVHLQEASRSSGRPEEELA       161
MRAP2 Rat      SDKVFSRQGNEESRSLFHCYINEVEHLDRVKVCHRTTAIDSDVHLQEAIRSSGRPEEELT       161
MRAP2 Cow      PDKVFSRQGNEESRSFFHCYINEVDHLEKAKACLQTTALDSEVHLQEAIRCSGRPEEELN       177
MRAP2 Dog      PDKVFSRQGNDESRSLFHCYINEVEHLDRAKVCHQTTVLDSSVRLQEAIRSNGRPEEELN       180
               *********:****:********::*::.*.* :**.:**.*:**** *..*:*****

MRAP2 Human    RLMKFDIPNFVNTDQN-YFGEDDLLISEPPIVLETKPLSQTSHKDLD                    205
MRAP2 Mouse    RFMKFDIPNFVNTEQS-SFGEDDLLISEAPVLLENKPVSQTSRIDLD                    207
MRAP2 Rat      RFMQFDIPNFVNTDQN-SFGEDDLLISEPPVLLENKPVSQTSRTDLD                    207
MRAP2 Cow      RLMKFDIPNFVNTDQN-SFGEDDLLISETPIVLENKPVVQTSHKELD                    223
MRAP2 Dog      RLMKFDIPNFVNTDQNSSFGEDDLLISEPPIVLENKPVSQTSHKDLD                    227
               *:*:*********:*.  ********** *::**.**: ***: :**


MC3R Human     MNASCCLPSVQPTLPNGSEHLQAPFFSNQSSSAFCEQVFIKPEVFLSLGIVSLLENILVI       60
MC3R Mouse     MNSSCCLSSVSPMLPNLSEHPAAPPASNRSGSGFCEQVFIKPEVFLALGIVSLMENILVI       60
MC3R Rat       MNSSCCPSSSYPTLPNLSQHPAAPSASNRSGSGFCEQVFIKPEVFLALGIVSLMENILVI       60
MC3R Cow       MNASCCLHSAQPKLPNSSEHVAAPSFSNQSSSRFCEQVFIKPEVFLALGIISLLENILVI       60
MC3R Dog       MNASCCLLSAQPTLPNSSEHLAAASFGNQSGSGFCEQVFIKPEVFLALGIVSLLENILVI       60
               **:***   *  * *** *:*  *   .*:*.* ************:***:**:******

MC3R Human     LAVVRNGNLHSPMYFFLCSLAVADMLVSVSNALETIMIAIVHSDYLTFEDQFIQHMDNIF       120
MC3R Mouse     LAVVRNGNLHSPMYFFLCSLAAADMLVSLSNSLETIMIAVINSDSLTLEDQFIQHMDNIF       120
MC3R Rat       LAVVRNGNLHSPMYFFLCSLAAADMLVSLSNSLETIMIVVINSDSLTLEDQFIQHMDNIF       120
MC3R Cow       LAVVRNGNLHSPMYFFLCSLAVADMLVSVSNALETIMIAVVNSDYLTLEDQFIQHMDNVF       120
MC3R Dog       LAVARNGNLHSPMYFFLCSLAVADMLVSVSNALETVMIAIVNSNYLTFEDRFVQHMDNVF       120
               ***.*****************.******:**:***:**.:::*: **:**:*:*****:*

MC3R Human     DSMICISLVASICNLLAIAVDRYVTIFYALRYHSIMTVRKALTLIVAIWVCCGVCGVVFI       180
MC3R Mouse     DSMICISLVASICNLLAIAIDRYVTIFYALRYHSIMTVRKALTLIGVIWVCCGICGVMFI       180
MC3R Rat       DSMICISLVASICNLLAIAVDRYVTIFYALRYHSIMTVRKALSLIVAIWVCCGICGVMFI       180
MC3R Cow       DSMICISLVASICNLLAIAVDRYVTIFYALRYHSIMTVRKALALIVAIWLGCGICGVVFI       180
MC3R Dog       DSMICISLVASICNLLAIAVDRYVTIFYALRYHSIMTVRKALAWIVAIWVCCGVCGVVFI       180
               *******************:**********************: * .**: **:***:**

MC3R Human     VYSESKMVIVCLITMFFAMMLLMGTLYVHMFLFARLHVKRIAALPPADGVAPQQHSCMKG       240
MC3R Mouse     IYSESKMVIVCLITMFFAMVLLMGTLYIHMFLFARLHVQRIAVLPPAGVVAPQQHSCMKG       240
MC3R Rat       VYSESKMVIVCLITMFFAMVLLMGTLYIHMFLFARLHVQRIAALPPADGVAPQQHSCMKG       240
MC3R Cow       VYSESKMVIVCLVTMFLAMLLLMGTLYVHMFLFARLHVKRIAALPPADGAAPQQHSCMKG       240
MC3R Dog       VYSESKMVIVCLITMFFAMLLLMGTLYVHMFLFARLHVQRIAALPPADGVAPQQHSCMKG       240
               :***********:***:**:*******:**********:***.****.  .*********

MC3R Human     AVTITILLGVFIFCWAPFFLHLVLIITCPTNPYCICYTAHFNTYLVLIMCNSVIDPLIYA       300
MC3R Mouse     AVTITILLGVFIFCWAPFFLHLVLIITCPTNPYCICYTAHFNTYLVLIMCNSVIDPLIYA       300
MC3R Rat       AVTITILLGVFIFCWAPFFLHLVLIITCPTNPYCICYTAHFNTYLVLIMCNSVIDPLIYA       300
MC3R Cow       AVTITILLGVFVFCWAPFFLHLVLIITCPTNPYCVCYTAHFNTYLVLIMCNSVIDPLIYA       300
MC3R Dog       AVTITILLGVFIFCWAPFFLHLVLIITCPTNPYCVCYTAHFNTYLVLIMCNSIIDPLIYA       300
               ***********:*********************:*****************:*******

MC3R Human     FRSLELRNTFREILCGCNGMNLG                                            323
MC3R Mouse     FRSLELRNTFKEILCGCNSMNLG                                            323
MC3R Rat       FRSLELRNTFKEILCGCNGMNVG                                            323
MC3R Cow       FRSLELRNTFKEILCSCNGMNLG                                            323
MC3R Dog       FRSLELRNTFKEILCSCNGMNLG                                            323
               **********:****.**.**:*
```

Fig. S9. Multiple sequence alignments of MRAP2 and MC3R in five mammalian species

Bolded residues that are shaded were predicted as contact sites and mutated in studies in this paper.

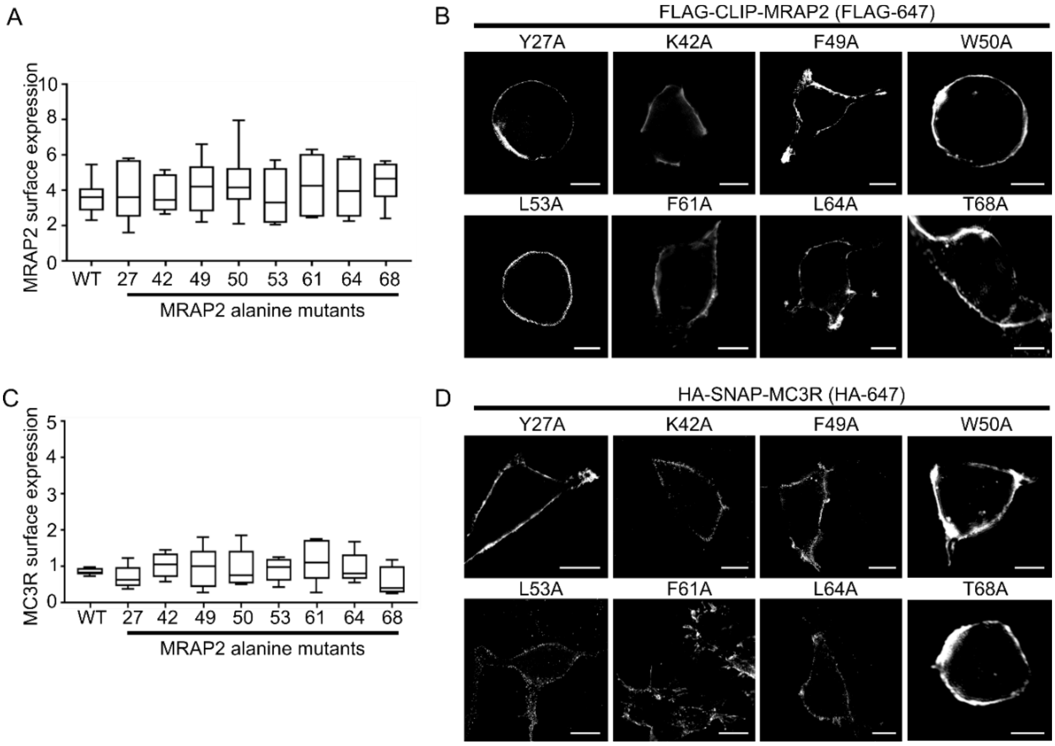

Fig. S10. Effect of MRAP2 alanine mutations on MRAP2 and MC3R expression

(**A**) Surface expression of MRAP2 in cells transfected with the FLAG-MRAP2 variants and incubated with FLAG antibody and anti-mouse Alexa Fluor 647. Values were normalized to pcDNA transfected cells. N=6-8 biological replicates per group. (**B**) SIM imaging of MRAP2 surface expression in non-permeabilized cells. Representative of 4 biological replicates per group. Scale bar, 5 µm. (**C**) Surface expression of MC3R in cells transfected with the HA-HALO-MC3R and FLAG-MRAP2 variants and incubated with HA antibody and anti-mouse Alexa Fluor 647. Values were normalized to pcDNA transfected cells. N=5 biological replicates per group. (**D**) SIM imaging of MC3R surface expression in non-permeabilized cells transfected with one of the eight alanine variants. Representative of 4 biological replicates per group. Scale, 5 µm. Statistical analyses were performed by one-way ANOVA with Sidak's multiple-comparisons test in (A) and (C).

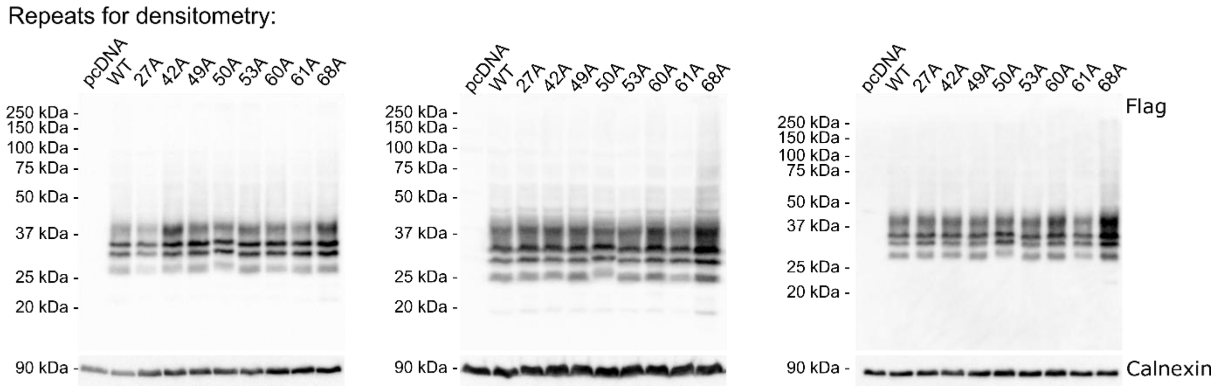

Fig. S11. Additional blots for the FLAG-MRAP2 alanine mutants quantified in Table S4

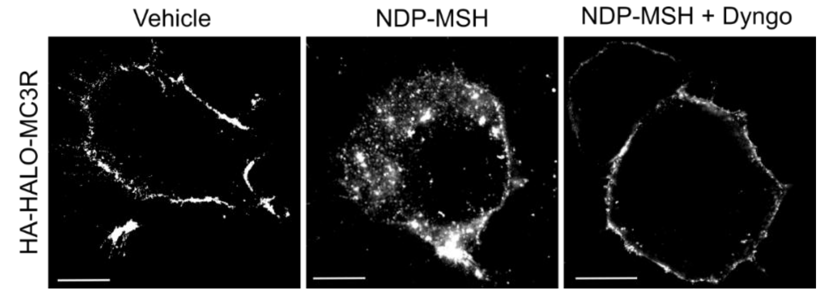

Fig. S12. Dyngo-4a blocks MC3R internalization
Cells expressing HA-HALO-MC3R were exposed to vehicle, NDP-MSH, or NDP-MSH and Dyngo-4a. Surface HA-HALO-MC3R in nonpermeabilized cells were visualized by immunofluorescence for HA. Representative of 6-8 images from each of 3 biological replicates per group. Scale bar, 5 µm.

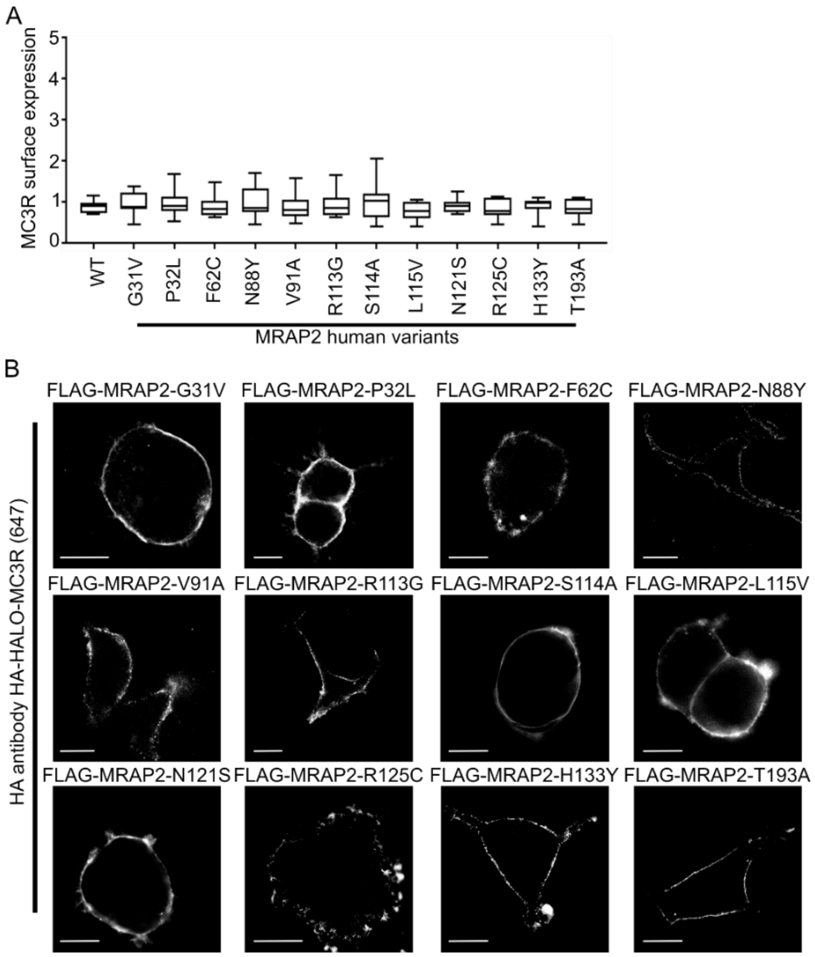

Fig. S13. Effect of MRAP2 human variants on MC3R cell surface expression
(**A**) Surface expression of MC3R in cells transfected with HA-HALO-MC3R and FLAG-MRAP2 variants and incubated with HA antibody and anti-mouse Alexa Fluor 647. Values were normalized to pcDNA transfected cells. N=10 biological replicates per group. (**B**) SIM imaging of MC3R surface expression in non-permeabilized cells. Representative of 4 biological replicates per group. Scale bar, 5 µm. Statistical analyses were performed by one-way ANOVA with Sidak's multiple-comparisons test in (A).

HA constructs:

Key: Secretory peptide HA tag Halo or SNAP tag

ATGGTCCTTCTGTTGATCCTGTCAGTCCTACTTCTGAAAGAAGATGTACGAGGGAGTGCACAGTCCACGCGATACCCCTACGACGTGCCCGACTACGCCACGCGTGGATCCGGATCC …tag… AGATCT …gene

MVLLLILSVLLLKEDVRGSAQSTRYPYDVPDYATRGSGS …tag… RS …gene

Flag constructs:

Key: Secretory peptide Flag tag CLIP tag

ATGGTCCTTCTGTTGATCCTGTCAGTCCTACTTCTGAAAGAAGATGTACGAGGGAGTGCACAGTCCACGCGAGATTATAAAGATGACGATGACAAAACGCGAGGATCC …tag… AGATCT

MVLLLILSVLLLKEDVRGSAQSTRPVDYKDDDDKTRGS …tag… RS …gene

Sequencing results for constructs:

>HA-Halo MC3R

TCCCAGGTCCAACTGCACCTCGGTTCTATCGATTGAATTCCTTTCCTAAAATGGTCCTTCTGTTGATCCTGTCAGTCCTACTTCTGAAAGAAGATGTACGAGGGAGTGCACAGTCCACGCGATACCCCTACGACGTGCCCGACTACGCCACGCGTGGATCCGGATCCGAAATCGGTACTGGCTTTCCATTCGACCCCCATTATGTGGAAGTCCTGGGCGAGCGCATGCACTACGTCGATGTTGGTCCGCGCGATGGCACCCCTGTGCTGTTCCTGCACGGTAACCCGACCTCCTCCTACGTGTGGCGCAACATCATCCCGCATGTTGCACCGACCCATCGCTGCATTGCTCCAGACCTGATCGGTATGGGCAAATCCGACAAACCAGACCTGGGTTATTTCTTCGACGACCACGTCCGCTTCATGGATGCCTTCATCGAAGCCCTGGGTCTGGAAGAGGTCGTCCTGGTCATTCACGACTGGAGCTCCGCTCTGGGTTTCCACTGGGCCAAGCGCAATCCAGAGCGCGTCAAAGGTATTGCATTTATGGAGTTCATCCGCCCTATCCCGACCTGGGACGAATGGCCAGAATTTGCCCGCGAGACCTTCCAGGCCTTCCGCACCACCGACGTCGGCCGCAAGCTGATCATCGATCAGAACGTTTTTATCGAGGGTACGCTGCCGATGGGTGTCGTCCGCCCGCTGACTGAAGTCGAGATGGACCATTACCGCGAGCCGTTCCTGAATCCTGTTGACCGCGAGCCACTGTGGCGCTTCCCAAACGAGCTGCCAATCGCCGGTGAGCCAGCGAACATCGTCGCGCTGGTCGAAGAATACATGGACTGGCTGCACCAGTCCCCTGTCCCGAAGCTGCTGTTCTGGGGCACCCCAGGCGTTCTGATCCCACCGGCCGAAGCCGCTCGCCTGGCCAAAAGCCTGCCTAACTGCAAGGCTGTGGACATCGGCCCGGGTTTGAATCTGCTGCAAGAAGACAACCCGGACCTGATCGGCAGCGAGATCGCGCGCTGGCTGTCTACTCTGGAGATTTCCGGTAGATCTAATGCTTCGTGCTGTTTGCCCTCAGTTCAGCCCACCCTCCCCAATGGCAGTGAGCATCTCCAGGCCCCTTTTTTCTCTAATCAAAGTTCATCTGCCTTCTGCGAACAGGTGTTCATCAAGCCAGAGGTGTTTCTGAGTCTGGGGATCGTGAGTCTCCTGGAGAACATTCTGGTGATCCTCGCGGTGGTACGGAACGGAAACCTGCACAGCCCAATGTACTTCTTTCTTTGCAGTCTGGCTGTGGCCGATATGCTGGTGTCAGTGTCCAACGCTCTGGAAACTATCATGATCGCTATCGTCCACTCAGACTATCTCACGTTCGAGGATCAGTTCATTCAACACATGGACAACATTTTTGATTCCATGATTTGCATCTCTCTCGTTGCTTCCATTTGCAATTTGTTGGCCATCGCCGTCGATCGGTACGTCACAATCTTTTACGCTTTGAGGTATCACTCCATCATGACCGTGCGGAAAGCACTCACACTGATTGTTGCAATCTGGGTCTGTTGTGGTGTGTGTGGCGTGGTTTTTATCGTTTATTCCGAGTCAAAGATGGTTATCGTGTGCTTGATCACTATGTTTTTCGCCATGATGCTTTTGATGGGAACACTGTATGTCCATATGTTCCTGTTTGCAAGACTGCATGTAAAACGAATCGCCGCCCTTCCTCCTGCAGACGGTGTCGCCCCCCAGCAGCATAGCTGCATGAAGGGGGCTGTGACTATAACAATCCTTCTGGGCGTTTTCATTTTCTGCTGGGCGCCATTTTTCCTCCATCTGGTCCTGATCATCACCTGCCCGACTAACCCGTATTGCATCTGCTACACCGCGCATTTCAATACGTACCTGGTCCTGATTATGTGTAACAGTGTTATAGATCCTTTGATTTATGCCTTCAGATCCCTGGAACTTAGAAACACATTTCGAGAGATTCTGTGTGGGTGTAACGGCATGAATCTGGGTTAGGTCGACCTGCAGAAGCTTGGCCGCCATGACCCAACTTGTTTATTGCAGCTTATAATGGTTACAAATAAAGCAATAGC

>HA-SNAP MC3R

TCCTAAAATGGTCCTTCTGTTGATCCTGTCAGTCCTACTTCTGAAAGAAGATGTACGAGGGAGTGCACAGTCCACGCGATACCCCTACGACGTGCCCGACTACGCCACGCGTGGATCCGACAAAGACTGCG

AAATGAAGCGCACCACCCTGGATAGCCCTCTGGGCAAGCTGGAACTGTCTGGGTGCGAACAGGGCCtGCACGAGATCAAGCTGCTGGGCAAAGGAACATCTGCCGCCGACGCCGTGGAAGTGCCTGCCCCAGCCGCCGTGCTGGGCGGACCAGAGCCACTGATGCAGGCCACCGCCTGGCTCAACGCCTACTTTCACCAGCCTGAGGCCATCGAGGAGTTCCCTGTGCCAGCCCTGCACCACCCAGTGTTCCAGCAGGAGAGCTTTACCCGCCAGGTGCTGTGGAAACTGCTGAAAGTGGTGAAGTTCGGAGAGGTCATCAGCTACCAGCAGCTGGCCGCCCTGGCCGGCAATCCCGCCGCCACCGCCGCCGTGAAAACCGCCCTGAGCGGAAATCCCGTGCCCATTCTGATCCCCTGCCACCGGGTGGTGTCTAGCTCTGGCGCCGTGGGGGGCTACGAGGGCGGGCTCGCCGTGAAAGAGTGGCTGCTGGCCCACGAGGGCCACAGACTGGGCAAGCCTGGGCTGGGCAGATCTAATGCTTCGTGCTGTTTGCCCTCAGTTCAGCCCACCCTCCCCAATGGCAGTGAGCATCTCCAGGCCCCTTTTTTCTCTAATCAAAGTTCATCTGCCTTCTGCGAACAGGTGTTCATCAAGCCAGAGGTGTTTCTGAGTCTGGGGATCGTGAGTCTCCTGGAGAACATTCTGGTGATCCTCGCGGTGGTACGGAACGGAAACCTGCACAGCCCAATGTACTTCTTTCTTTGCAGTCTGGCTGTGGCCGATATGCTGGTGTCAGTGTCCAACGCTCTGGAAACTATCATGATCGCTATCGTCCACTCAGACTATCTCACGTTCGAGGATCAGTTCATTCAACACATGGACAACATTTTTGATTCCATGATTTGCATCTCTCTCGTTGCTTCCATTTGCAATTTGTTGGCCATCGCCGTCGATCGGTACGTCACAATCTTTTACGCTTTGAGGTATCACTCCATCATGACCGTGCGGAAAGCACTCACACTGATTGTTGCAATCTGGGTCTGTTGTGGTGTGTGTGGCGTGGTTTTTATCGTTTATTCCGAGTCAAAGATGGTTATCGTGTGCTTGATCACTATGTTTTTCGCCATGATGCTTTTGATGGGAACACTGTATGTCCATATGTTCCTGTTTGCAAGACTGCATGTAAAACGAATCGCCGCCCTTCCTCCTGCAGACGGTGTCGCCCCCCAGCAGCATAGCTGCATGAAGGGGGCTGTGACTATAACAATCCTTCTGGGCGTTTTCATTTTCTGCTGGGCGCCATTTTTCCTCCATCTGGTCCTGATCATCACCTGCCCGACTAACCCGTATTGCATCTGCTACACCGCGCATTTCAATACGTACCTGGTCCTGATTATGTGTAACAGTGTTATAGATCCTTTGATTTATGCCTTCAGATCCCTGGAACTTAGAAACACATTTCGAGAGATTCTGTGTGGGTGTAACGGCATGAATCTGGGTTAGGTCGACCTGCAGAAGCTTGGCCgccatgtcccaaCTTGTTTATTGCAGCTTATAATGGTTACAAATAAAgCAATAGcaTCacAAATTCAC

>Flag-CLIP MC3R

GGTCCAACTGCACCTCGGTTCTATCGATTGAATTCCTTTCCTAAAATGGTCCTTCTGTTGATCCTGTCAGTCCTACTTCTGAAAGAAGATGTACGAGGGAGTGCACAGTCCACGCGACCGGTAGATTATAAAGATGACGATGACAAAACGCGAGGATCCGACAAGGATTGTGAAATGAAACGCACCACACTGGACAGCCCTTTGGGGAAGCTGGAGCTGTCTGGTTGTGAGCAGGGTCTGCACGAAATAATTTTTCTGGGCAAGGGGACGTCTGCAGCTGATGCCGTGGAGGTCCCAGCCCCCGCTGCGGTTCTCGGAGGTCCGGAGCCCCTGATACAGGCTACAGCCTGGCTGAATGCCTATTTCCACCAGCCCGAGGCTATCGAAGAGTTCCCCGTGCCGGCTCTTCACCATCCCGTTTTCCAGCAAGAGTCGTTCACCAGACAGGTGTTATGGAAGCTGCTGAAGGTTGTGAAATTCGGAGAAGTGATTTCTGAGTCACACTTAGCAGCCCTGGTAGGCAACCCCGCAGCCACGGCAGCAGTGAATACGGCACTGGATGGCAATCCTGTCCCTATCCTGATCCCGTGCCACAGAGTGGTCCAGGGGGATTCGGATGTGGGGCCGTACCTTGGTGGACTGGCCGTGAAGGAATGGCTTCTGGCCCATGAAGGCCACCGGTTGGGGAAGCCAGGCTTGGGAAGATCTAATGCTTCGTGCTGTTTGCCCTCAGTTCAGCCCACCCTCCCCAATGGCAGTGAGCATCTCCAGGCCCCTTTTTTCTCTAATCAAAGTTCATCTGCCTTCTGCGAACAGGTGTTCATCAAGCCAGAGGTGTTTCTGAGTCTGGGGATCGTGAGTCTCCTGGAGAACATTCTGGTGATCCTCGCGGTGGTACGGAACGGAAACCTGCACAGCCCAATGTACTTCTTTCTTTGCAGTCTGGCTGTGGCCGATATGCTGGTGTCAGTGTCCAACGCTCTGGAAACTATCATGATCGCTATCGTCCACTCAGACTATCTCACGTTCGAGGATCAGTTCATTCAACACATGGACAACATTTTTGATTCCATGATTTGCATCTCTCTCGTTGCTTCCATTTGCAATTTGTTGGCCATCGCCGTCGATCGGTACGTCACAATCTTTTACGCTTTGAGGTATCACTCCATCATGACCGTGCGGAAAGCACTCACACTGATTGTTGCAATCTGGGTCTGTTGTGGTGTGTGTGGCGTGGTTTTTATCGTTTATTCCGAGTCAAAGATGGTTATCGTGTGCTTGATCACTATGTTTTTCGCCATGATGCTTTTGATGGGAACACTGTATGTCCATATGTTCCTGTTTGCAAGACTGCATGTAAAACGAATCGCCGCCCTTCCTCCTGCAGACGGTGTCGCCCCCCAGCAGCATAGCTGCATGAAGGGGGCTGTGACTATAACAATCCTTCTGGGCGTTTTCATTTTCTGCTGGGCGCCATTTTTCCTCCATCTGGTCCTGATCATCACCTGCCCGACTAACCCGTATTGCATCTGCTACACCGCGCATTTCAATACGTACCTGGTCCTGATTATGTGTAACAGTGTTATAGATCCTTTGATTTATGCCTTCAGATCCCTGGAACTTAGAAACACATTTCGAGAGATTCTGTGTGGGTGTAACGGCATGAATCTGGGTTAGGTCGACCTGCAGAAGCTTGGCCGCCTTGGCCCAACTTGTTTATTGCAGCTTATAATGGTTACAAATAAAGCAATAGCATCACAAATT

>HA-Halo MRAP2

GTCCNCTCCCAGGTCCAACTGCACCTCGGTTCTATCGATTGAATTCCTTTCCTAAAATGGTCCTTCTGTTGATCCTGTCAGTCCTACTTCTGAAAGAAGATGTACGAGGGAGTGCACAGTCCACGCGATACCCCTACGACGTGCCCGACTACGCCACGCGTGGATCCGGATCCGAAATCGGTACTGGCTTTCCATTCGACCCCCATTATGTGGAAGTCCTGGGCGAGCGCATGCACTACGTCGATGTTGGTCCGCGCGATGGCACCCCTGTGCTGTTCCTGCACGGTAACCCGACCTCCTCCTACGTGTGGCGCAACATCATCCCGCATGTTGCACCGACCCATCGCTGCATTGCTCCAGACCTGATCGGTATGGGCAAATCCGACAAACCAGACCTGGGTTATTTCTTCGACGACCACGTCCGCTTCATGGATGCCTTCATCGAAGCCCTGGGTCTGGAAGAGGTCGTCCTGGTCATTCACGACTGGGGCTCCGCTCTGGGTTTCCACTGGGCCAAGCGCAATCCAGAGCGCGTCAAAGGTATTGCATTTATGGAGTTCATCCGCCCTATCCCGACCTGGGACGAATGGCCAGAATTTGCCCGCGAGACCTTCCAGGCCTTCCGCACCACCGACGTCGGCCGCAAGCTGATCATCGATCAGAACGTTTTTATCGAGGGTACGCTGCCGATGGGTGTCGTCCGCCCGCTGACTGAAGTCGAGATGGACCATTACCGCGAGCCGTTCCTGAATCCTGTTGACCGCGAGCCACTGTGGCGCTTCCCAAACGAGCTGCCAATCGCCGGTGAGCCAGCGAACATCGTCGCGCTGGTCGAAGAATACATGGACTGGCTGCACCAGTCCCCTGTCCCGAAGCTGCTGTTCTGGGGCACCCCAGGCGTTCTGATCCCACCGGCCGAAGCCGCTCGCCTGGCCAAAAGCCTGCCTAACTGCAAGGCTGTGGACATCGGCCCGGGTCTGAATCTGCTGCAAGAAGACAACCCGGACCTGATCGGCAGCGAGATCGCGCGCTGGCTGTCTACTCTGGAGATTTCCGGTAGATCTTCCGCCCAGAGGTTAATTTCTAACAGAACCTCCCAGCAATCGGCATCTAATTCTGATTACACCTGGGAATATGAATATTATGAGATTGGACCAGTTTCCTTTGAAGGACTGAAGGCTCATAAATATTCCATTGTGATTGGATTTTGGGTTGGTCTTGCAGTCTTCGTGATTTTTATGTTTTTGTGCTGACCTTGCTGACCAAGACAGGAGCCCCACACCAAGACAATGCAGAGTCCTCAGAGAAGAGATTCAGAATGAACAGCTTTGTGTCAGACTTTGGAAGACCTCTGGAGCCAGATAAAGTATTTTCTCGCCAAGGCAACGAGGAGTCCAGGTCTCTCTTTCACTGCTACATCAATGAGGTGGAACGCTTGGACAGAGCCAAAGCTTGTCACCAGACCACAGCCCTTGACAGTGACGTCCAACTCCAGGAAGCCATCAGAAGCAGTGGGCAGCCAGAGGAGGAGCTGAACAGGCTCATGAAGTTTGACATCCCCAACTTTGTGAACACAGACCAGAACTACTTTGGGGAGGATGATCTTCTGATTTCTGAACCACCTATTGTTCTGGAAACTAAGCCACTTTCCCAGACCTCACACAAAGACCTGGATTAGGTCGACCTGCAGAAGCTTGGCCGCCATGGCCCAACTTGTTTATTGCAGCTTATAATGGTTACAAATAAAGCAATAGCATCACAA

>HA-SNAP MRAP2

ACCGGGGACCGATCCAGCCTCCGCggccGGGAaCGGTgcATTGGAACGCGGATTCCCCGTGCCAAGAGTGACGTAAGTACCGCCTATAGAGTCTATAGGCCCACCCCCTTGGCTTCGTTAGAACGCGGCTACaATTAATACATAACCTTATGTATCATACACATACGATTTAGGTGACACTATAGAATAACATCCACTTTGCCTTTCTCTCCACAGGTGTCCACTCCCAGGTCCAACTGCACCTCGGTTCTATCGATTGAATTCCTTTCCTAAAATGGTCCTTCTGTTGATCCTGTCAGTCCTACTTCTGAAAGAAGATGTACGAGGGAGTGCACAGTCCACGCGATACCCCTACGACGTGCCCGACTACGCCACGCGTGGATCCGACAAAGACTGCGAAATGAAGCGCACCACCCTGGATAGCCCTCTGGGCAAGCTGGAACTGTCTGGGTGCGAACAGGGCCtGCACGAGATCAAGCTGCTGGGCAAAGGAACATCTGCCGCCGACGCCGTGGAAGTGCCTGCCCCAGCCGCCGTGCTGGGCGGACCAGAGCCACTGATGCAGGCCACCGCCTGGCTCAACGCCTACTTTCACCAGCCTGAGGCCATCGAGGAGTTCCCTGTGCCAGCCCTGCACCACCCAGTGTTCCAGCAGGAGAGCTTTACCCGCCAGGTGCTGTGGAAACTGCTGAAAGTGGTGAAGTTCGGAGAGGTCATCAGCTACCAGCAGCTGGCCGCCCTGGCCGGCAATCCCGCCGCCACCGCCGCCGTGAAAACCGCCCTGAGCGGAAATCCCGTGCCCATTCTGATCCCCTGCCACCGGGTGGTGTCTAGCTCTGGCGCCGTGGGGGGCTACGAGGGCGGGCTCGCCGTGAAAGAGTGGCTGCTGGCCCACGAGGGCCACAGACTGGGCAAGCCTGGGCTGGGCAGATCTTCCGCCCAGAGGTTAATTTCTAACAGAACCTCCCAGCAATCGGCATCTAATTCTGATTACACCTGGGAATATGAATATTATGAGATTGGACCAGTTTCCTTTGAAGGACTGAAGGCTCATAAATATTCCATTGTGATTGGATTTTGGGTTGGTCTTGCAGTCTTCGTGATTTTTATGTTTTTGTGCTGACCTTGCTGACCAAGACAGGAGCCCCACACCAAGACAATGCAGAGTCCTCAGAGAAGAGATTCAGAATGAACAGCTTTGTGTCAGACTTTGGAAGACCTCTGGAGCCAGATAAAGTATTTTCTCGCCAAGGCAACGAGGAGTCCAGGTCTCTCTTTCACTGCTACATCAATGAGGTGGAACGCTTGGACAGAGCCAAAGCTTGTCACCAGACCACAGCCCTTGACAGTGACGTCCAACTCCAGGAAGCCATCAGAAGCAGTGGGCAGCCAGAGGAGGAGCTGAACAGGCTCATGAAGTTTGACATCCCCAACTTTGTGAACACAGACCAGAACTACTTTGGGGAGGATGATCTTCTGATTTCTGAACCACCTATTGTTCTGGAAACTAAGCCACTTTCCCAGACCTCACACAAAGACCTGGATTAGGTCGACCTGCAGAAGCTTGGCCGCCATGGCCCAACTTGTTTATTGCAGCTTATAATGGTTACAAATAAAGCAATAGCATCACAAATTCACAAATAAA

>Flag-CLIP MRAP2

GACCTCCATAGAAGACACCGGGACCGATCCAGCCTCCGCGGCCGGGAACGGTGCATTGGAACGCG
GATTCCCCGTGCCAAGAGTGACGTAAGTACCGCCTATAGAGTCTATAGGCCCACCCCCTTGGCTTC
GTTAGAACGCGGCTACAATTAATACATAACCTTATGTATCATACACATACGATTTAGGTGACACTA
TAGAATAACATCCACTTTGCCTTTCTCTCCACAGGTGTCCACTCCCAGGTCCAACTGCACCTCGGTT
CTATCGATTGAATTCCTTTCCTAAAATGGTCCTTCTGTTGATCCTGTCAGTCCTACTTCTGAAAGAA
GATGTACGAGGGAGTGCACAGTCCACGCGACCGGTAGATTATAAAGATGACGATGACAAAACGCG
AGGATCCGACAAGGATTGTGAAATGAAACGCACCACACTGGACAGCCCTTTGGGGAAGCTGGAGC
TGTCTGGTTGTGAGCAGGGTCTGCACGAAATAATTTTTCTGGGCAAGGGGACGTCTGCAGCTGATG
CCGTGGAGGTCCCAGCCCCCGCTGCGGTTCTCGGAGGTCCGGAGCCCCTGATACAGGCTACAGCCT
GGCTGAATGCCTATTTCCACCAGCCCGAGGCTATCGAAGAGTTCCCCGTGCCGGCTCTTCACCATC
CCGTTTTCCAGCAAGAGTCGTTCACCAGACAGGTGTTATGGAAGCTGCTGAAGGTTGTGAAATTCG
GAGAAGTGATTTCTGAGTCACACTTAGCAGCCCTGGTAGGCAACCCCGCAGCCACGGCAGCAGTG
AATACGGCACTGGATGGCAATCCTGTCCCTATCCTGATCCCGTGCCACAGAGTGGTCCAGGGGGAT
TCGGATGTGGGGCCGTACCTTGGTGGACTGGCCGTGAAGGAATGGCTTCTGGCCCATGAAGGCCA
CCGGTTGGGGAAGCCAGGCTTGGGAAGATCTTCCGCCCAGAGGTTAATTTCTAACAGAACCTCCCA
GCAATCGGCATCTAATTCTGATTACACCTGGGAATATGAATATTATGAGATTGGACCAGTTTCCTT
TGAAGGACTGAAGGCTCATAAATATTCCATTGTGATTGGATTTTGGGTTGGTCTTGCAGTCTTCGTG
ATTTTTATGTTTTTTGTGCTGACCTTGCTGACCAAGACAGGAGCCCCACACCAAGACAATGCAGAG
TCCTCAGAGAAGAGATTCAGAATGAACAGCTTTGTGTCAGACTTTGGAAGACCTCTGGAGCCAGA
TAAAGTATTTTCTCGCCAAGGCAACGAGGAGTCCAGGTCTCTCTTTCACTGCTACATCAATGAGGT
GGAACGCTTGGACAGAGCCAAAGCTTGTCACCAGACCACAGCCCTTGACAGTGACGTCCAACTCC
AGGAAGCCATCAGAAGCAGTGGGCAGCCAGAGGAGGAGCTGAACAGGCTCATGAAGTTTGACAT
CCCCAACTTTGTGAACACAGACCAGAACTACTTTGGGGAGGATGATCTTCTGATTTCTGAACCACC
TATTGTTCTGGAAACTAAGCCACTTTCCCAGACCTCACACAAAGACCTGGATTAGGTCGACCTGCA
GAAGCTTGGCCGCCATGGCCCAACTTGTTTATTGCAGCTTATAATGGTTACAAATAAAGCAATAGC

Fig. S14. Plasmid sequence data for MC3R and MRAP2

Table S1. Top 15 MC3R-positive and top 15 MC4R-positive clusters in the human HYPOMAP dataset

			% cells/ cluster			Average log normalized expression/cluster			% coexpressing cells/ cluster	
**Cluster name**	**Predicted region**	**Top marker genes**	***MRAP2***	***MC3R***	***MC4R***	***MRAP2***	***MC3R***	***MC4R***	***MC3R & MRAP2***	***MC4R & MRAP2***
C4-390 Mid-2 GABA-GLU-3 PGR TAC1	ARC	KISS1|NR5A2|SKOR2|PGR	46.94	27.76	20.82	0.41	0.17	0.12	16.73	10.61
C4-388 Mid-2 GABA-GLU-3 PGR CALCR	ARC	PGR|CALCR|NR5A2|LHX4	37.06	23.08	4.9	0.12	0.06	0.01	12.59	2.8
C4-64 Mid-1 GABA-1 NR5A2	Periventricular	SLC6A3|NR5A2|DLK1|MC3R	19.23	21.15	1.92	0.21	0.31	0.03	4.81	0
C4-391 Mid-2 GABA-GLU-3 PGR TAC3	ARC	KISS1|SKOR2|UGT2B7|TAC3	26.82	14.8	15.08	0.21	0.08	0.11	6.7	5.59
C4-161 Mid-1 GABA-6 IL13RA1 GHRH	ARC	GHRH|GAL|ADGRF4|GHSR	8.56	12.83	0	0.07	0.1	0	0.53	0
C4-345 Mid-2 GLU-2 ARHGAP42 COL15A1	VMH	NR5A1|COL15A1|FEZF1|CCBE1	27.8	11.5	12.99	0.23	0.09	0.13	5.32	6.07
C4-375 Mid-2 GABA-GLU-3 POMC ANKRD30A	ARC	SOX3|PGR|POMC|TBX3	69.75	10.92	16.25	0.65	0.04	0.09	8.96	14.01
C4-136 Mid-1 GABA-5 GAL PGR	ARC	GAL|MBNL3|NTS|PGR	24.3	8.41	11.53	0.16	0.06	0.08	1.87	2.49
C4-376 Mid-2 GABA-GLU-3 PDGFD PGR	ARC	PGR|GABRE|ALDH1A1|VGLL3	26.05	7.78	11.08	0.24	0.02	0.06	3.89	5.99
C4-374 Mid-2 GABA-GLU-3 POMC CALCR	ARC	CALCR|POMC|WIF1|PGR	33.69	7.28	2.7	0.27	0.03	0.01	4.31	1.35
C4-349 Mid-2 GLU-2 SLC22A10 CCBE1	VMH	SLC22A10|PGR|DNAH11|ADAMTSL1	50	6.8	9.71	0.45	0.05	0.07	4.37	4.85
C4-385 Mid-2 GABA-GLU-3 PGR DNAH11	ARC	SKOR2|KISS1|DNAH11|VGLL3	22.17	6.6	17.45	0.14	0.02	0.08	2.83	5.66
C4-387 Mid-2 GABA-GLU-3 PGR TRPC6	ARC	PGR|TBX3|PGR-AS1|NR5A2	45.37	6.32	19.19	0.28	0.03	0.11	2.71	10.38
C4-207 Pre-2 GABA-4 SATB2 TNS3	MPOA	SATB2|COL15A1|EGFLAM|ZIC2	39.51	5.85	5.37	0.38	0.03	0.05	4.88	2.44
C4-76 Mid-1 GABA-2 CLMP MBNL3	SCN	NR2F2-AS1|ARHGAP36|SP9|Z96074.1	63.19	5.56	4.17	0.43	0.02	0.03	5.56	2.08
C4-303 Mid-3 GLU-3 SLITRK6 FBN2	MAM	FBN2|TACR3|SIM1|OTP	52.68	0	41.07	0.28	0	0.23	0	25
C4-144 Mid-1 GABA-5 RORB GLI3	MPOA	GLI3|TPTE|GAL|HMX3	58.11	0	31.08	0.49	0	0.23	0	21.62
C4-328 Mid-2 GLU-1 SOX14 CYP19A1	MPOA	SKOR2|CYP19A1|FAM9B|QRFPR	56.45	0	27.96	0.39	0	0.18	0	17.2
C4-194 Pre-2 CHOL-1 BMPR1B	LPOA	SLC5A7|COL6A5|CHAT|LHX8	37.7	0	27.16	0.29	0	0.31	0	13.42
C4-306 Mid-3 GLU-3 CD36 LMCD1	MAM	OTP|KCNH8|LMCD1|STK32B	52.9	0	26.09	0.46	0	0.2	0	15.22
C4-206 Pre-2 GABA-4 LHX6 NR0B1	NA	NPY|SHISAL2B|NR0B1|LHX6	66.23	0	24.68	0.57	0	0.13	0	20.78
C4-396 Mid-2 GLU-4 WNT7B PRRX1	NA	LEF1|WIF1|RSPO3|TRABD2B	18.02	0	22.52	0.12	0	0.15	0	5.41
C4-406 Mid-2 GLU-4 EYA4 NPNT	TMN	WIF1|LEF1|TBX3|VEGFC	33.2	3.86	21.24	0.23	0.01	0.13	2.32	10.81
C4-18 Pre-1 GABA-1 SEMA3C CAV1	NA	CAV1|PROK2|CXCL14|CALCRL	16.67	0	20.6	0.28	0	0.27	0	4.86
C4-171 Pre-2 GABA-1 IL1RAPL2 SST	LPOA	MOXD1|IL1RAPL2|LHX6|NXPH2	62.34	0	20.08	0.81	0	0.21	0	16.32
C4-29 Pre-1 GABA-1 PRKCH PWWP3B	NA	TAC3|SLC22A10|SCML4|EBF1	82.29	0	20	0.77	0	0.1	0	17.14
C4-382 Mid-2 GABA-GLU-3 LEF1 IL1RAPL2	TMN	LEF1|IL1RAPL2|CCN3|ANKRD30A	47.25	0	19.72	0.37	0	0.12	0	11.01
C4-348 Mid-2 GLU-2 SLC22A10 WDR64	VMH	NR2F2-AS1|SLC22A10|SATB1-AS1|CCBE1	44.53	2.73	19.53	0.43	0.03	0.13	0.78	12.11
C4-118 Mid-1 GABA-4 NTS ABCG2	MPOA	TMEM114|ESR1|FAM9B|PGR	78.88	0	19.4	0.7	0	0.08	0	18.53

ARC, arcuate nucleus; MPOA, medial preoptic area; NA, nucleus accumbens; SCN, suprachiasmatic nucleus; TMN, Tuberomammillary nucleus; VMH, ventromedial nucleus of the hypothalamus.

Table S2. Expression plasmids

Plasmid name		Information		Source
cAMP Glosensor-22F		cAMP sensor		Promega
ss-HA-Halo-MC3R		N-terminal signal peptide (MVLLLILSVLLLKEDVRG) from mGluR5, followed by HA, HALO and human MC3R		This manuscript
ss-HA-SNAP-MC3R		N-terminal signal peptide (MVLLLILSVLLLKEDVRG) from mGluR5, followed by HA, SNAP and human MC3R		This manuscript
ss-FLAG-CLIP-MC3R		N-terminal signal peptide (MVLLLILSVLLLKEDVRG) from mGluR5, followed by HA, CLIP and human MC3R		This manuscript
ss-HA-Halo-MRAP2		N-terminal signal peptide (MVLLLILSVLLLKEDVRG) from mGluR5, followed by HA, Halo and human MRAP2		This manuscript
ss-HA-SNAP-MRAP2		N-terminal signal peptide (MVLLLILSVLLLKEDVRG) from mGluR5, followed by HA, SNAP and human MRAP2		This manuscript
ss-FLAG-CLIP-MRAP2		N-terminal signal peptide (MVLLLILSVLLLKEDVRG) from mGluR5, followed by HA, CLIP and human MC3R		This manuscript
ss-HA-Halo-mGluR2		Used as template for ss-HA-Halo-MC3R		Joshua Levitz, Weill Cornell Medicine
ss-HA-SNAP-mGluR2		Used as template for ss-HA-SNAP-MC3R		Joshua Levitz, Weill Cornell Medicine
ss-FLAG-CLIP-mGluR2		Used as template for ss-FLAG-CLIP-MC3R		Joshua Levitz, Weill Cornell Medicine
ss-HA-Halo-MC4R		N-terminal signal peptide from mGluR5, followed by HA, HALO and human MC3R		Caroline Gorvin, University of Birmingham
ss-FLAG-CLIP-SSTR3		N-terminal signal peptide from mGluR5, followed by HA, HALO and human SSTR3		This manuscript
SSTR3-Tango		Used as a template for SSTR3 constructs		Addgene plasmid #66504
MC3R-Tango		Used as a template for MC3R constructs		Addgene plasmid #66429
MRAP2-3xFLAG		Used as a template for MRAP2 constructs and in most assays		Julien Sebag, University of Iowa
MRAP2 untagged		Native full-length MRAP2 with no tags in pcDNA3.1 backbone		This manuscript
MC3R untagged		Native full-length MC3R with no tags in pcDNA3.1 backbone		This manuscript
B-arrestin2-mYFP		SIM		Addgene plasmid #36917
Nluc-Arr2		BRET		Steve Hill, University of Nottingham
Venus-Kras		BRET		Nevin Lambert, Augusta University
Rab5-Venus		SIM		Nevin Lambert, Augusta University
LgC-MC3R		NanoBiT		This manuscript
SmC-MC3R		NanoBiT		This manuscript
LgC-MRAP2		NanoBiT		This manuscript
SmC-MRAP2		NanoBiT		This manuscript
MRAP2-3xFLAG-Y27A		Glosensor, SIM, cell surface expression		This manuscript
MRAP2-3xFLAG-K42A		Glosensor, SIM, cell surface expression		Caroline Gorvin, University of Birmingham
MRAP2-3xFLAG-F49A		Glosensor, SIM, cell surface expression		This manuscript
MRAP2-3xFLAG-W50A		Glosensor, SIM, cell surface expression		This manuscript
MRAP2-3xFLAG-L53A		Glosensor, SIM, cell surface expression		This manuscript
MRAP2-3xFLAG-F61A		Glosensor, SIM, cell surface expression		This manuscript
MRAP2-3xFLAG-L64A		Glosensor, SIM, cell surface expression		Caroline Gorvin, University of Birmingham
MRAP2-3xFLAG-T68A		Glosensor, SIM, cell surface expression		Caroline Gorvin, University of Birmingham
ss-HA-Halo-MC3R-T245A		Glosensor, SIM, cell surface expression		This manuscript
ss-HA-Halo-MC3R-L260A		Glosensor, SIM, cell surface expression		This manuscript
ss-HA-Halo-MC3R-P272A		Glosensor, SIM, cell surface expression		This manuscript
MRAP2-3xFLAG-G31V		Glosensor, SIM, cell surface expression		Caroline Gorvin, University of Birmingham

MRAP2-3xFLAG-P32L		Glosensor, SIM, cell surface expression		Caroline Gorvin, University of Birmingham
MRAP2-3xFLAG-F62C		Glosensor, SIM, cell surface expression		Caroline Gorvin, University of Birmingham
MRAP2-3xFLAG-N88Y		Glosensor, SIM, cell surface expression		Caroline Gorvin, University of Birmingham
MRAP2-3xFLAG-V91A		Glosensor, SIM, cell surface expression		Caroline Gorvin, University of Birmingham
MRAP2-3xFLAG-R113G		Glosensor, SIM, cell surface expression		Caroline Gorvin, University of Birmingham
MRAP2-3xFLAG-S114A		Glosensor, SIM, cell surface expression		Caroline Gorvin, University of Birmingham
MRAP2-3xFLAG-L115V		Glosensor, SIM, cell surface expression		Caroline Gorvin, University of Birmingham
MRAP2-3xFLAG-N121S		Glosensor, SIM, cell surface expression		Caroline Gorvin, University of Birmingham
MRAP2-3xFLAG-R125C		Glosensor, SIM, cell surface expression		Caroline Gorvin, University of Birmingham
MRAP2-3xFLAG-H133Y		Glosensor, SIM, cell surface expression		Caroline Gorvin, University of Birmingham
MRAP2-3xFLAG-T193A		Glosensor, SIM, cell surface expression		Caroline Gorvin, University of Birmingham

Table S3. Identification of possible contacts between MRAP2 and MC3R in AlphaFold2 models

		**MC3R**				
**MRAP2**	**Region[#]**	**Rank 1**	**Rank 2**	**Rank 3**	**Rank 4**	**No. of models[$]**
**Trp^{23}**	EC/IC		Asn^{68}			1
**Glu^{26}**	EC/IC	Asp^{117}				1
**Tyr^{27}**	EC/IC	Phe^{281}	Lys^{239}, Gly^{240}	Asp^{121}	Tyr^{299}, Arg^{302}	**4**
**Ile^{30}**	EC/IC		Gln^{233}, His^{235}		Ser^{236}	2
**Lys^{39}**	EC/IC	Phe^{34}				1
**Ala^{40}**	EC/IC	Tyr^{273}	Met^{238}			2
**Lys^{42}**^	TM	Pro^{272}, Tyr^{273}		Met^{187}		2
**Tyr^{43}**	TM		Val^{242}		Met^{238}	2
**Ser^{44}**	TM		Phe^{211}			1
**Ile^{45}**	TM			Met^{187}		1
**Val^{46}**	TM			Val^{190}	Thr^{245}	2
**Phe^{49}**	TM	Leu^{260}		Cys^{191}, Met^{195}	Gly^{249}	**3**
**Trp^{50}**^	TM	Pro^{257}	Thr^{245}, Leu^{248}, Gly^{249}		Leu^{248}	**3**
**Leu^{53}**^	TM	Ala^{256}		Ala^{198}	Ile^{252}	**3**
**Phe^{61}**^	TM		Val^{263}	Thr^{205}	Phe^{197}	**3**
**Leu^{64}**^	TM	Thr^{245}	Leu^{260}	Leu^{206}	Leu^{260}	**4**
**Leu^{67}**	TM	Phe^{211}, Arg^{215}				1
**Thr^{68}**	TM		Thr^{267}	His^{209}		**2**
**Phe^{90}**	IC/EC		Asp^{117}			1
**Arg^{113}**	IC/EC	Pro^{225}				1
**Ile^{120}**	IC/EC			Gln^{223}		1
**Phe^{169}**	IC/EC	Ser^{236}				1

[#]Region refers to the structural location as either: EC, extracellular region, TM, transmembrane helix, IC, intracellular region. Some residues are designated as IC/EC because the orientation of MRAP2 differs in some models (Fig. S8A-B). [$]No. of models refers to the total number (out of 4) of structural models that identified a link between the MRAP2 residue and MC3R. MRAP2 and MC3R residues that were mutated to alanine and functionally characterized in this study are in red and blue, respectively. [^]Alanine substitutions that impair MRAP2 enhancement of MC3R signaling (Figs. 4F, 4I, 4J, 4L, and 4N).

Table S4. Densitometry of FLAG-MRAP2 protein with alanine variants

Human MRAP2 (relative to WT)	Mean±SEM (N)
Y27A	1.13 ± 0.03 (4)
K42A	1.04 ± 0.06 (4)
F49A	0.97 ± 0.06 (4)
W50A	1.11 ± 0.03 (4)
L53A	0.93 ± 0.07 (4)
F61A	1.04 ± 0.09 (4)
L64A	0.93 ± 0.09 (4)
T68A	1.68 ± 0.19 (4)****
pcDNA	0.01 ± 0.01 (4)****

Densitometry of FLAG-MRAP2 protein from the four Western blots in Fig. 4B and S11. MRAP2 variants were investigated in separate batches and protein expression was normalized to the MRAP2-WT control in each experiment. Asterisks indicate significant difference to WT. ****p<0.0001 compared to MRAP2-WT by one-way ANOVA with Dunnett's multiple comparisons test.

Table S5. pEC_{50} and E_{max} values for cAMP production from Fig. 4 for MRAP2 alanine mutations at predicted interaction sites

		**MRAP2-WT**	**MRAP2 variant**	**pcDNA**
**Y27A**	**pEC_{50} ± SEM**	7.55 ± 0.44	7.82 ± 0.37	7.95 ± 0.51
	**E_{max} ± SEM**	166.3 ± 16.13**	165.4 ± 14.05**	100
**K42A**	**pEC_{50} ± SEM**	8.61 ± 0.33	8.60 ± 0.57	8.86 ± 0.33
	**E_{max} ± SEM**	224.4 ± 16.57****	123.9 ± 7.37****	100
**F49A**	**pEC_{50} ± SEM**	8.25 ± 0.36	8.46 ± 0.32	8.68 ± 0.33
	**E_{max} ± SEM**	179.3 ± 19.94*	169.5 ± 21.45*	100
**W50A**	**pEC_{50} ± SEM**	7.37 ± 0.16	7.18 ± 0.52	7.86 ± 0.42
	**E_{max} ± SEM**	183.1 ± 15.14*	113.7 ± 21.41*	100
**L53A**	**pEC_{50} ± SEM**	8.39 ± 0.47	7.80 ± 0.72	8.75 ± 0.39
	**E_{max} ± SEM**	190.8 ± 22.09**	126.6 ± 25.22*	100
**F61A**	**pEC_{50} ± SEM**	7.37 ± 0.16	7.19 ± 0.94	7.86 ± 0.42
	**E_{max} ± SEM**	183.1 ± 15.4*	89.83 ± 26.68*	100
**L64A**	**pEC_{50} ± SEM**	8.50 ± 0.39	8.79 ± 0.27	8.68 ± 0.33
	**E_{max} ± SEM**	200.7 ± 17**	126.8 ± 29.7*	100
**T68A**	**pEC_{50} ± SEM**	8.12 ± 0.51	7.65 ± 0.29	7.79 ± 0.07
	**E_{max} ± SEM**	222.2 ± 13.54**	231.1 ± 27.68***	100

Statistical analyses were performed by one-way ANOVA with Tukeys's test. Black asterisks show comparisons to pcDNA. Blue asterisks compare MRAP2-variant with MRAP2-WT.

Table S6. pEC_{50} and E_{max} values for cAMP from Fig. 5 for MC3R alanine mutations at predicted interaction sites

		MC3R-WT + pcDNA	MC3R-WT + MRAP2	MC3R-mutant + pcDNA	MC3R-mutant + MRAP2
**T245A**	**pEC_{50} ± SEM**	8.73 ± 0.34	9.04 ± 0.31	8.18 ± 0.21	8.59 ± 0.53
	**E_{max} ± SEM**	100*	172.2 ± 22.68	106.1 ± 5.91*	102.6 ± 12.51**
**T260A**	**pEC_{50} ± SEM**	8.89 ± 0.28	8.90 ± 0.33	9.06 ± 0.30	8.21 ± 0.44
	**E_{max} ± SEM**	100*	178.2 ± 22.4	57.53 ± 14.33***	101 ± 9.23
**P272A**	**pEC_{50} ± SEM**	8.56 ± 0.36	9.01 ± 0.31	8.30 ± 0.37	8.45 ± 0.29
	**E_{max} ± SEM**	100**	173.1 ± 12.68	106.6 ± 10.15*	106.3 ± 14.65*

Statistical analyses were performed by one-way ANOVA with Tukeys's test for pEC_{50} values and Kruskal-Wallis with Dunn's test for E_{max} values. Black asterisks compare MC3R-WT with MRAP2.

Table S7. pEC_{50} and E_{max} values for β-arrestin from Fig. 7 for MRAP2 alanine mutations at predicted interaction sites

		MRAP2-WT	MRAP2 variant	pcDNA
**Y27A**	**pEC_{50} ± SEM**	7.81 ± 0.28	7.93 ± 0.52	7.20 ± 0.22
	**E_{max} ± SEM**	54.55 ± 6.21****	65.39 ± 5.39***	100
**K42A**	**pEC_{50} ± SEM**	7.81 ± 0.28	7.68 ± 0.47	7.20 ± 0.22
	**E_{max} ± SEM**	54.55 ± 6.21***	102.5 ± 10.42***	100
**F49A**	**pEC_{50} ± SEM**	7.77 ± 0.38	7.99 ± 0.47	7.37 ± 0.16
	**E_{max} ± SEM**	53.14 ± 6.33****	47.01 ± 6.30****	100
**W50A**	**pEC_{50} ± SEM**	7.81 ± 0.28	8.35 ± 0.23**	7.20 ± 0.22
	**E_{max} ± SEM**	54.55 ± 6.21***	97.6 ± 10.17***	100
**L53A**	**pEC_{50} ± SEM**	7.81 ± 0.28	7.58 ± 0.57	7.20 ± 0.22
	**E_{max} ± SEM**	54.55 ± 6.21****	95.97 ± 7.73***	100
**F61A**	**pEC_{50} ± SEM**	7.81 ± 0.28	8.16 ± 0.32*	7.20 ± 0.22
	**E_{max} ± SEM**	54.55 ± 6.21****	96.6 ± 5.01****	100
**L64A**	**pEC_{50} ± SEM**	7.77 ± 0.38	7.30 ± 0.23	7.37 ± 0.16
	**E_{max} ± SEM**	54.20 ± 6.25****	100.6 ± 1.36****	100
**T68A**	**pEC_{50} ± SEM**	7.77 ± 0.38	8.17 ± 0.38	7.37 ± 0.16
	**E_{max} ± SEM**	54.20 ± 6.25**	59.94 ± 14.81*	100

Statistical analyses were performed by one-way ANOVA with Tukeys's test. Black asterisks show comparisons to pcDNA. Blue asterisks compare MRAP2-variant with MRAP2-WT.

Table S8. pEC$_{50}$ and E$_{max}$ values for β-arrestin from Fig. 7 for MC3R alanine mutations at predicted interaction sites

		MC3R-WT + pcDNA	MC3R-WT + MRAP2	MC3R-mutant + pcDNA	MC3R-mutant + MRAP2
**T245A**	**pEC$_{50}$ ± SEM**	9.17 ± 0.30	9.67 ± 0.41	9.69 ± 0.46	8.86 ± 0.43
	**E$_{max}$ ± SEM**	100**	56.18 ± 9.11	106.43 ± 10.19**	101.14 ± 11.00**
**T260A**	**pEC$_{50}$ ± SEM**	9.17 ± 0.30	9.67 ± 0.41	8.04 ± 0.44*	8.45 ± 0.40
	**E$_{max}$ ± SEM**	100**	56.18 ± 9.11	49.07 ± 10.80	72.21 ± 11.72
**P272A**	**pEC$_{50}$ ± SEM**	9.17 ± 0.30	9.67 ± 0.41	8.64 ± 0.62	8.63 ± 0.56
	**E$_{max}$ ± SEM**	100**	56.18 ± 9.11	113.11 ± 15.11***	106.86 ± 6.52**

Statistical analyses were performed by one-way ANOVA with Tukeys's test for pEC$_{50}$ values and Kruskal-Wallis with Dunn's test for E$_{max}$ values. Black asterisks compare MC3R-WT with MRAP2.

Table S9. Identification of possible contacts between MC3R and MRAP2 residues mutated in overweight and/or obese individuals

Variant	Rank 1	Rank 2	Rank 3	Rank 4
**G31V**	Glu^{29}	None	Val^{33}	None
**P32L**	Ser^{34}	None	Ser^{34}	None
**F62C**	None	Ile^{58}, Leu^{66}	None	None
**N88Y**	None Mutant forms new contact with Ser^{89}.	Arg^{84}, Ser^{92}. Mutant forms new contacts with Phe^{85}, Arg^{86}.	None	Ser^{92} Mutant forms new contact with Arg^{84}.
**V91A**	None	None	None	Phe^{94}
**R113G**	Link to MC3R Pro^{225}. Mutant loses contact.	None	Glu^{111}	None
**S114A**	Link to MC3R Asn^{159}. Mutant loses contact.	None	None	None
**L115V**	None	None	None	None
**N121S**	His^{117}, Cys^{118}, Arg^{125}, Asn^{171}	None	His^{117}, Cys^{118}, Arg^{125}, Glu^{124}. Mutant loses His^{117} contact.	None
**R125C**	Asn^{121}, Ala^{129}	None	Asn^{121}, Glu^{122}	None
**H133Y**	Lys^{130}	None	Lys^{130} Mutant loses contact.	None
**T193A**	None	None	None	None

Contacts are in MRAP2 and are intramolecular unless otherwise stated. The predicted consequences of MRAP2 variants are highlighted in red.
